# Observational studies generate misleading results about the health effects of air pollution: Evidence from chronic air pollution and COVID-19 outcomes

**DOI:** 10.1371/journal.pone.0296154

**Published:** 2024-01-02

**Authors:** Marc N. Conte, Matthew Gordon, Nicole A. Swartwood, Rachel Wilwerding, Chu A. (Alex) Yu

**Affiliations:** 1 Department of Economics, Fordham University, Bronx, NY, United States of America; 2 Paris School of Economics, Paris, France; 3 Department of Global Health and Population, Harvard T.H. Chan School of Public Health, Boston, MA, United States of America; 4 Department of Economics, Wake Forest University, Winston-Salem, NC, United States of America; Wuhan University, CHINA

## Abstract

Several observational studies from locations around the globe have documented a positive correlation between air pollution and the severity of COVID-19 disease. Observational studies cannot identify the causal link between air quality and the severity of COVID-19 outcomes, and these studies face three key identification challenges: 1) air pollution is not randomly distributed across geographies; 2) air-quality monitoring networks are sparse spatially; and 3) defensive behaviors to mediate exposure to air pollution and COVID-19 are not equally available to all, leading to large measurement error bias when using rate-based COVID-19 outcome measures (e.g., incidence rate or mortality rate). Using a quasi-experimental design, we explore whether traffic-related air pollutants cause people with COVID-19 to suffer more extreme health outcomes in New York City (NYC). When we address the previously overlooked challenges to identification, we do not detect causal impacts of increased chronic concentrations of traffic-related air pollutants on COVID-19 death or hospitalization counts in NYC census tracts.

## Introduction

Policymakers and researchers seek to understand the drivers of differential impacts of environmental and public health hazards to provide targeted aid to the most vulnerable populations. Misunderstandings about the factors that cause these hazards to impose severe impacts can lead to ineffective policy and distrust or skepticism about information coming from official sources.

Traffic-related air pollutants (TRAPs) are a major cause of poor local air quality in many parts of the world. The relationship between air particulates and respiratory and cardiac function has long been accepted. As such, air pollution is recognized as imposing major costs on society via mortality, morbidity, and other effects [1–3].

PM_2.5_, and NO_x_ are TRAPs with known health hazards that have been the focus of numerous studies, generally observational in nature, exploring the relationship between air quality conditions and COVID-19 disease severity [4–20]. Table 1 summarizes a surveyed set of these studies, which have generally found positive correlations between worse chronic air quality and the severity of COVID-19 disease globally [13] and at the county level in the United States [14]. The exception is [16], which found a negative correlation.

**Table 1:** Summary of surveyed studies.

Study	Study Region and Time Period	Spatial Unit of Pollution Estimation	Spatial Unit of COVID-19 Outcome Aggregation	Modeled Effect Based on Reported Results	Challenge I: Endogenous Concentration Measures	Challenge II: Measurement Error in Pollutant Concentrations	Challenge III: Measurement Error in Dependent Variables
**Hospitalizations**							
Frontera et al., (2020)	Italy, Feb-Mar 31, 2020 (n = 10,572)	Region	Region	4.735	Not addressed	Highly Susceptible	Count
Bowe et al., (2021)	USA, Mar 2, 2020-Jan 1, 2021, (n = 169,102)	1 km^2^ area encircling patient address	Individual patient	0.402	Not addressed	Susceptible	Rate/ratio
Mendy et al., (2021)	University of Cincinnati Hospital System, Mar 13-Jul 5, 2020, (n = 1128)	Zip Code	Individual patient	4.735	Not addressed	Susceptible	Rate/ratio
Austin et al., (2023)	USA, Jan 22-Aug 15, 2020, (n = 97885)	County	County	0.061	Addressed	Highly Susceptible	Rate/ratio
**ICU Admissions**							
Frontera et al., (2020)	Italy, Feb-Mar 31, 2020, (n = 10,572)	Region	Region	4.966	Not addressed	Highly Susceptible	Count
Bozack et al., (2022)	NYC, Mar 8-Aug 30, 2020, (n = 6542)	100 m^2^ area encircling patient address	Individual patient	0.993	Not addressed	Susceptible	Rate/ratio
Austin et al., (2023)	USA, Jan 22-Aug 15, 2020, (n = 97885)	County	County	0.038	Addressed	Highly Susceptible	Rate/ratio
**Mortality**							
Bianconi, et al., (2020)	Italy, March 1-March 31, 2020, (n = 110)	Province	Province	5.193	Not addressed	Highly Susceptible	Rate/ratio
Frontera et al., (2020)	Italy, Feb-Mar 31, 2020, (n = 10,572)	Region	Region	4.048	Not addressed	Highly Susceptible	Count
Konstantinoudis et al., (2020)	England, Jan 1-June 30, 2020, (n = 38, 573)	1 km^2^ area encircling patient address	Lower Tier Local Authority	0.107	Not addressed	Susceptible	Rate/ratio
Liang, et al., (2020)	USA, Jan 22-Jul 17, 2020, (n = 3122)	County	County	0.438	Not addressed	Highly Susceptible	Rate/ratio
Petroni et al., (2020)	USA, Jan - May 13, 2020 (n = 3223)	County	County	0.535	Not addressed	Highly Susceptible	Rate/ratio
Pozzer et al., (2020)	Global, Available data as of 3rd week of June, 2020 (n = variable*)	Country	Country	0.611	Not addressed	Highly Susceptible	Rate/ratio
Wu, et al., (2020)	USA, Jan 1-Jun 18, 2020, (n = 3089)	County	County	0.840	Not addressed	Highly Susceptible	Rate/ratio
Jiang and Xu, (2021)	Wuhan, China, Jan 25-April 7, 2020, (n = 73)	City	City	0.604	Not addressed	Highly Susceptible	Count
Kim and Bell, (2021)	NYC, Feb 29, 2020-Jan 5, 2021, (n = 177)	Neighborhood	Neighborhood	-0.382	Not addressed	Susceptible	Rate/ratio
Meo, et al., (2021)	California, Mar 19-Sept 22, 2020, (n = 1878)	County	County	0.671	Not addressed	Highly Susceptible	Count
Meo, et al., (2021)	Los Angeles, New Mexico, New York, Ohio, and Florida, Mar 13-Dec 31, 2020, (n = 1465)	Region	Region	0.000	Not addressed	Highly Susceptible	Count
Pansini and Fornacca, (2021)	China, Dec 19, 2019–May 23, 2020, (n = 31)	Province	Province	1.375	Not addressed	Highly Susceptible	Rate/ratio
Austin et al., (2023)	USA, Jan 22-Aug 15, 2020, (n = 97885)	County	County	0.306	Addressed	Highly Susceptible	Rate/ratio
**Case fatality**							
Yao, et al., (2020)	China, Jan 15- Mar 22, 2020, (n = 49)	City	City	0.003	Not addressed	Highly Susceptible	Rate/ratio
Bozack et al., (2022)	NYC, Mar 8-Aug 30, 2020, (n = 6542)	100 m^2^ area encircling patient addresses	Individual patient	0.840	Not addressed	Susceptible	Rate/ratio

Notes: This table presents summaries of recent observational studies exploring the relationships between PM2.5 and COVID-19 outcomes. Several of these studies examine associations between multiple pollutants and COVID-19 outcomes, however, in this table we document results for outcomes associated with PM2.5. The observational studies identified for potential inclusion in Table 1 were chosen because they had received coverage from major media outlets (e.g., [42]); they had a substantial number of citations, according to Google Scholar; or because they had been included in a highly-cited review of papers researching the links between air pollution and COVID-19 outcomes [43]. Table 1 includes a subset of these studies that report results on the relationship between PM2.5 and a COVID-19 outcome, such as the number of deaths, hospitalizations, mortality rates, etc. Studies examining the relationship between PM2.5 and the spread of COVID-19 (e.g. number of cases or infection rate) are excluded from the table, as are those primarily focused on pollutants other than PM2.5. The studies are categorized by the type of COVID-19 outcome and presented in chronological order. The modeled effects shown in column 5 are presented as elasticities, representing the percent change in the measured COVID-19 outcomes per percent change in PM2.5 concentrations. For uniformity, we use the average annual PM2.5 concentration in the US for 2019 (~7.64 ug/m3) as a baseline and use the deviation described in each paper (e.g. 1 ug/m3 increase) to calculate the percentage change in PM2.5 concentrations corresponding with each paper’s outcome of interest. All findings reported in this table are statistically significant (95% CI) except for the regional Meo et al. (2021) paper which finds no statistically significant relationship between PM2.5 and mortality.

* Pozzer et al. (2020) use country-level populations in mortality rate ratio calculations.

Determining whether increased air-pollutant concentrations cause adverse health effects is challenging for three reasons. First, air pollution is not randomly distributed across geographies. In fact, air quality and the health effect of interest (COVID-19 outcomes in this case) might both be highly correlated with unobservable community and demographic characteristics, preventing observational studies from identifying causal effects. Of the studies listed in Table 1, only [7] addresses the correlation between raw measures of air quality and demographic characteristics by employing a quasi-experimental design similar to the one that we apply here.

Second, air-quality monitoring networks are sparse spatially. Researchers studying the effects of air pollution on human capital usually measure pollution exposure by creating a proxy variable using nearby monitors recording ambient concentrations, which unavoidably induces measurement error that could bias coefficient estimates in unknown directions. This measurement error is a larger problem with data from spatially-sparse monitoring networks, which, under certain interpolation schemes, can smooth concentration data across communities, an issue referred to as the ecological fallacy.

Third, defensive behaviors to mediate exposure to air pollution and COVID-19 are not equally available to all. Thus, rate-based COVID-19 outcome measures (e.g., incidence rate or mortality rate), which depend on administrative data for population estimates, lead to systematic bias in assessing the health effects of air pollution. Existing research has shown that adoption of defensive behavior to limit exposure to air pollutants is associated with demographic characteristics that might also affect both the physiological and social response to COVID-19, including education [21], age, and insurance status [22]. In addition, the initial outbreak of COVID-19 caused many people to leave cities, which tend to have higher pollution levels. Failure to account for differential availability of defensive measures, including the ability to temporarily move away from infection hotspots, can bias regression results from both observational and causal studies.

To address these causal identification challenges, we employ a quasi-experimental design that uses an instrumental-variables (IV) approach and detailed air-quality data from New York City (NYC), the one-time global epicenter of the pandemic [23], to identify the causal relationship between chronic air-quality conditions and the intensity of COVID-19 disease. Our work highlights the challenges facing researchers from the fields of economics, public health, and other disciplines interested in providing policy guidance about the causal effects of air pollution offers through: 1) the use of hyperlocal air-quality data; 2) census-tract level data on COVID-19 outcomes; 3) econometric techniques to causally identify the variation in air quality that is independent of unobservable community characteristics; and 4) cellphone mobility data to highlight concerns about rate-based models dependent on administrative data for population estimates that ignore potential defensive behaviors.

We find that exogenous variation in the chronic ambient concentrations of our focal pollutants does not cause significant changes in COVID-19 outcomes. Our null effects are more precisely estimated for those pollutants most closely associated with traffic (NO and NO_2_), though we are unable to reject moderate effects even for these pollutants. These results contrast with the large correlations reported in a number of observational studies that are confounded by correlations between ambient pollutant concentrations and demographic characteristics that also affect health outcomes. We only replicate the findings from these observational studies of a substantial, positive correlation when we use a sample in which our instrument fails the exclusion restriction (S7 and S8 Tables in S1 File). In this case, our measure of air quality is similar to the raw pollutant concentrations used in the observational studies reported in Table 1, in that it is correlated with other determinants of COVID-19 outcomes. The implication being that reported *associations* between air quality and COVID-19 outcomes in this sample, and in the observational studies we survey, cannot be interpreted as *causal* effects, making it challenging to know if, and how, such results should impact public-health policy and individual behavior.

## Challenges to causal identification

Work exploring links between ambient pollutant concentrations and health outcomes, including COVID-19 outcomes, such as existing observational studies, is usually confronted by three challenges. Table 1 summarizes 17 representative studies, of which only [7] is quasi-experimental in nature, that have been referenced in major media outlets or academic reviews, and indicates which, if any, of these challenges are addressed in each study. In this section, we provide an in-depth discussion of the identification challenges facing this strand of the literature and replicate the results from existing studies that ignore these key identification issues. It is worth noting that none of the papers included in Table 1 address more than one of the challenges described below.

### Challenge 1: “Cleaning” endogenous air pollutant measures

Air pollution is correlated with many socioeconomic variables that may influence COVID-19 outcomes. It has been well documented that low-income and majority non-White neighborhoods are often subjected to higher concentrations of air pollutants [24–29], suggesting that poverty or other correlated characteristics could confound the results from recent observational studies about the relationship between air quality and COVID-19 outcomes [30]. These endogenous measures of air pollution will bias all coefficient estimates in such regressions [31], due to the causal link between air pollution and several individual characteristics (e.g., income, pre-existing health conditions). Importantly, it is difficult to know *a priori* the direction of the bias—correlational studies could either over or underestimate the effect of pollution on health. Some of the listed studies try to address this endogeneity concern by including demographic controls in the models, yet we show that adding such control variables would not solve the issue [32].

To overcome this challenge, we use an IV approach that relies on variation in pollution resulting from wind direction relative to nearby highways to identify the causal effects of chronic ambient air pollution on COVID-19 disease intensity. Our method compares tracts within the same neighborhood and same distance from a highway that only differ in the fraction of time spent downwind of the highway. Such tracts should be comparable on both observable and unobservable characteristics, while differing in their chronic air quality, so that we can isolate the effect of pollution on COVID-19 from the effects of other demographic characteristics. The key assumption underlying this method is that wind direction is exogenous to, or uncorrelated with, individual or community characteristics that are correlated with COVID-19 outcomes. In other words, wind direction only effects COVID-19 outcomes through its effect on air quality. Details of the IV approach are in the Methods section.

### Challenge 2: Constructing pollution exposure proxies with spatially-sparse monitoring networks

Aggregated ambient pollutant concentrations are typically used as a proxy for exposure, a particularly strong assumption given the coarse spatial resolution of most pollutant concentration measures, whether derived from sparse governmental monitoring networks or satellite estimates. Note that existing studies have tended to use county-level pollutant data aggregated or calibrated using EPA monitors, which usually bear the issues of being sparsely and strategically sited, resulting in known limitations of the EPA monitoring network and concentration estimates derived from satellite measurements [32–35]. A few studies listed in Table 1 [5, 8, 10] attempt to address such measurement issues by interpolating ambient pollution exposure to the agents’ location, which would only alleviate the measurement error concern when monitoring networks are densely sited [8]. However, in the more common case of having only one monitor within a county, every agent would be assigned the same pollution concentration regardless.

A more robust solution is to use air pollution data from a spatially-dense network of monitors, such as the New York City Community Air Survey (NYCCAS) monitoring network used in this paper (also used in [8] and [16]). The NYCCAS monitoring network provides fine-scale and high-quality pollution measurements at about 100 city locations in NYC between 2009–2018, as compared to the 12 EPA monitors in the city. We re-run our estimated models using pollutant concentrations constructed from the EPA monitors and find a much larger, though statistically insignificant, treatment effect than when we use pollutant concentrations constructed from the NYCCAS monitors.

NYC is one of the few places in the United States with such a concentrated network of monitors, and our results suggest that there is great value in the establishment of a national network at similar scales. Additionally, our data on census tract level COVID-19 outcomes allow for much more precise attribution of exposure than county-level data. Our results, which vary from those reported in several observational studies, highlight the importance of improved indoor and outdoor pollutant monitoring to acknowledge differential access to defensive behaviors across segments of our society.

### Challenge 3: Acknowledging that defensive behaviors threaten validity of rate-based models of COVID-19 outcomes

Studies relying on administrative population data to construct estimates of mortality and hospitalization rates during pandemics are subject to systematic measurement error in their estimated rates. This error in rate-based measures is a substantial problem for previous studies on this topic, as COVID-19 caused many people to leave cities, which tend to have higher pollution levels than rural and suburban areas. Many NYC residents fled the city in the spring of 2020 to avoid exposure to SARS-CoV-2, behavior that was reported to be concentrated in the city’s wealthiest neighborhoods [36]. This fact likely contributes to the results reported in [16] of a negative correlation between ambient pollutant concentrations and COVID-19 outcomes, because Manhattan saw large decreases in population that were not captured in administrative data and this borough tends to have high ambient pollutant concentrations.

Failure to account for differential availability of defensive measures, including the ability to temporarily move away from infection hotspots, can bias regression results from both observational and causal studies. Most of the studies listed in Table 1 use rate-based measures, without adjusting for migrations during the pandemic. Further, the standard practice of using population weights to account for heteroskedasticity in these rate-based models exacerbates the existing measurement error problem.

We incorporate cell phone mobility data from Safegraph to examine variation in defensive behavior. Safegraph collects location data from 45 million mobile devices and provides aggregated statistics at the census block group level on the amount of time that mobile devices are in the home, outside of the home, and engaged in work behavior, as well as distance travelled. We use this data to identify changes in the count of devices calling each census tract home during the spring of 2020.

**Fig 1:**
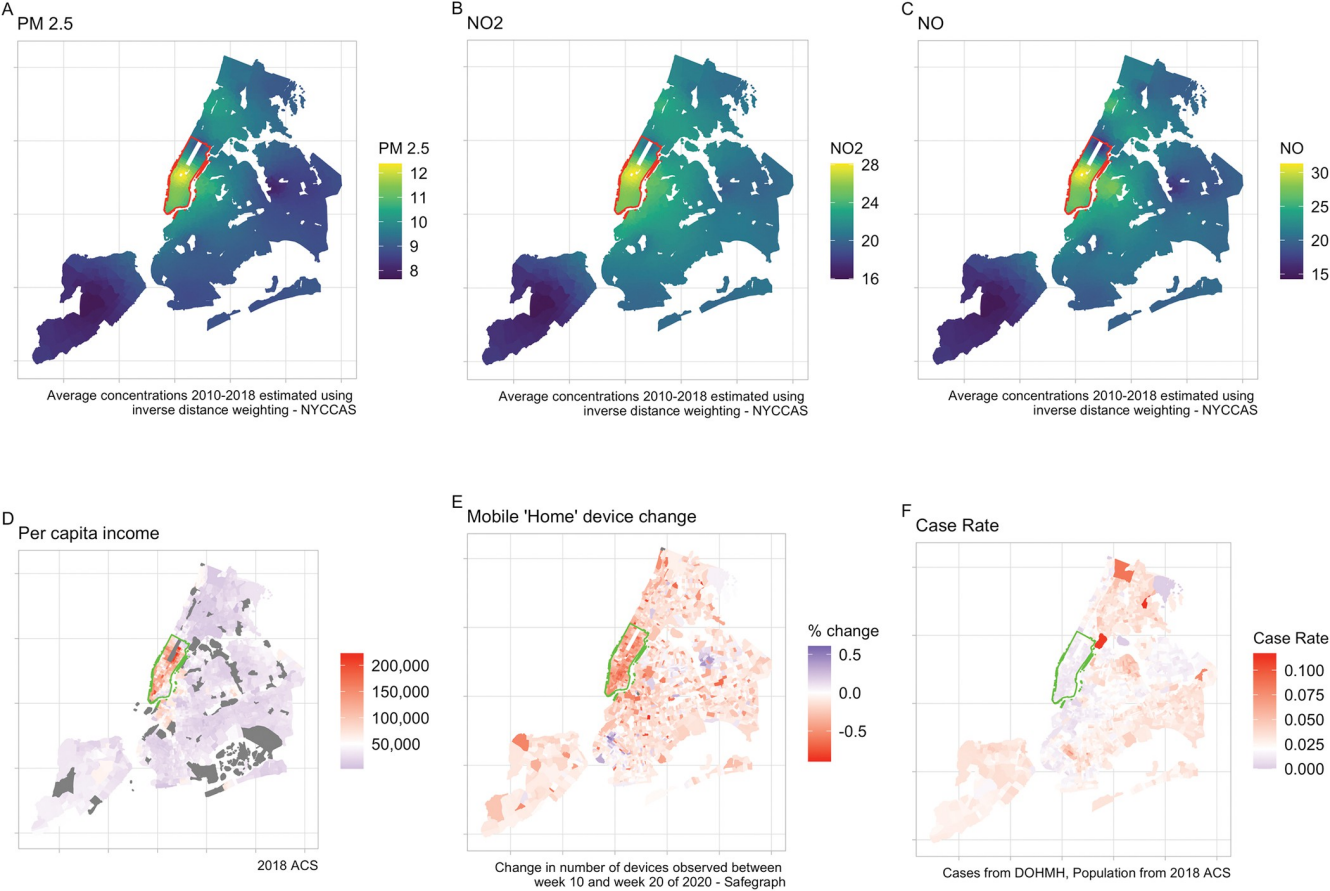
This figure depicts tract-level characteristics relating to chronic concentrations of PM_2.5_ (μg/m3) (A), NO_2_ (ppb) (B), NO (ppb) (C); 2018 per-capita income (D); the change in mobile devices based in each tract between week 10 and 20 of 2020 (E); and the case rate (positive test results divided by population) as of 08/31/2020 (F).

Fig 1 shows that while pollutant concentrations are highest in the wealthier parts of Manhattan with a larger fraction of White residents (panels A-C), the estimated case rate, defined as the number of positive tests divided by the census tract population (based on administrative data), is substantially lower for this part of the city (panel F). This may be due to increased adoption of defensive behaviors in these areas, as the Safegraph data shows a significant decrease in the number of cell phone devices residing in these census tracts during the first wave of the pandemic, consistent with individuals in these neighborhoods leaving the city to avoid exposure to the disease (panel E).

**Fig 2:**
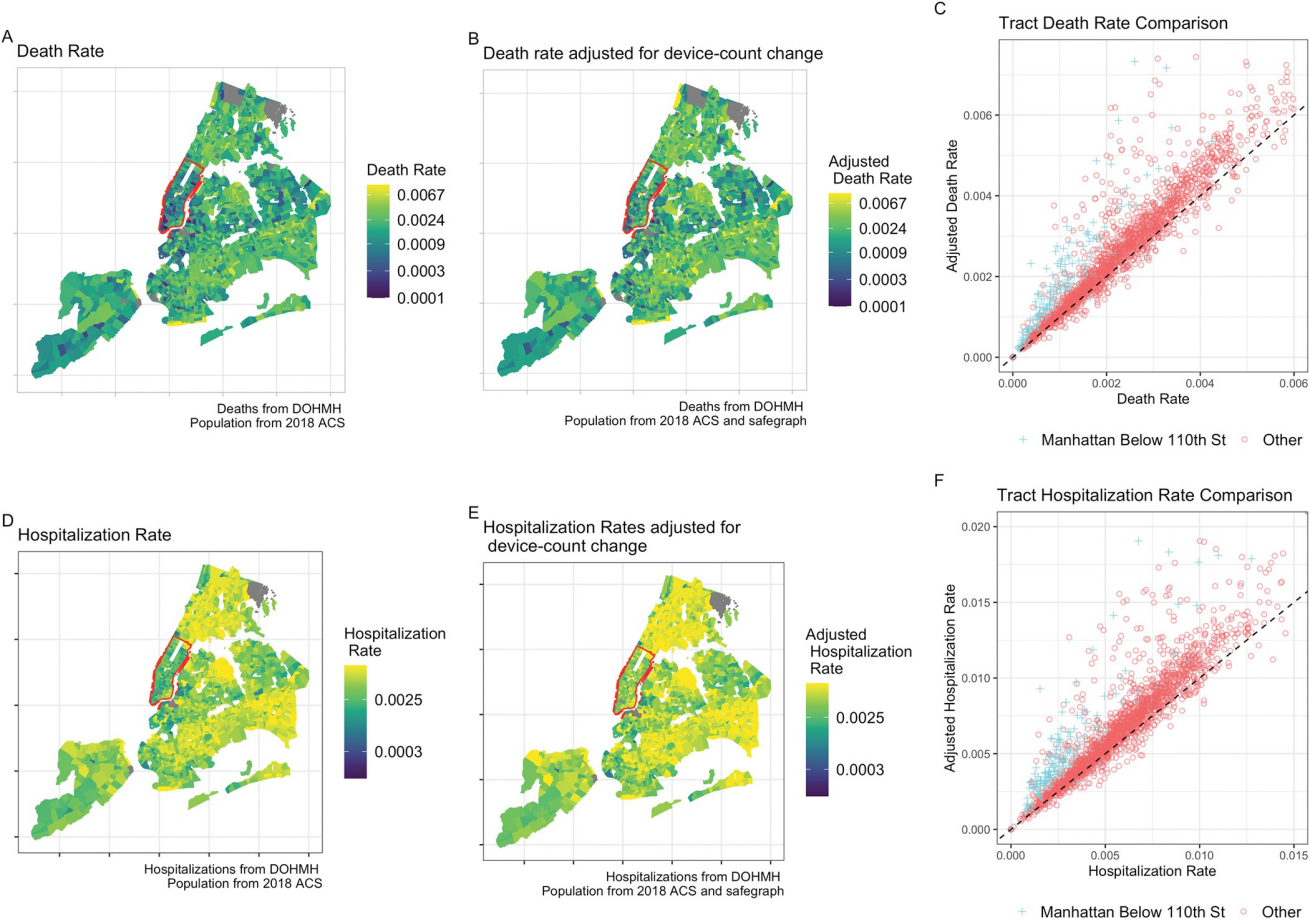
Measures of COVID-19 death and hospitalization rates based on different measures of census tract population. Safegraph adjusted death (B) and hospitalization rates (D) in Manhattan below 110^th^ Street are more comparable to those in the rest of the city, emphasizing how the inequitable access to defensive behaviors (namely relocating during the initial phase of the COVID-19 pandemic) can explain much of the observed gap in COVID-19 outcomes across different demographic groups in NYC.

We incorporate the Safegraph mobility data to adjust the rate-based outcome variables that have been used in previous studies. Fig 2 displays COVID-19 death and hospitalization rates, where these rates are calculated using different population estimates. Panels A and D of Fig 2 show these rates using population figures from the 2018 American Community Survey (ACS) in the denominator, and panels B and E use this population adjusted for the number of devices that left the city between March 8 and May 11, 2020 (weeks 10 and 20 of the year), which aligns with the peak of the initial wave of COVID-19 in NYC. Blue points in the scatter plots (panels C and F) show that adjusting these rate-based measures using cellphone mobility data results in much higher death and hospitalization rates in Manhattan relative to the rest of the city. A rough attempt to account for these departures would effectively double death rates in this part of Manhattan, making them more comparable to those in the rest of the city.

While the spatial patterns of air quality and demographic characteristics in NYC differ from other parts of the United States, our findings reinforce the point that disparities exist across incomes and racial groups regarding the ability to mitigate exposure to environmental and public health hazards, a point emphasized by a recent study of ambient air quality conditions in California [37]. The Safegraph data suggest that the wealthier parts of Manhattan below 110^th^ Street with a larger fraction of White residents was not less susceptible to COVID-19 or air pollution, but appeared to experience lower death and hospitalization rates than other groups largely due to defensive behaviors and temporary relocation that were not reflected in administrative data.

## Methods

Our preferred specifications use log-transformed counts of hospitalizations and deaths and an Instrumental Variable (IV) estimation method that includes two stages of regressions. In the first stage, we explain the variation in chronic TRAP concentrations that results from being downwind of a highway. We use the 10-year average pollutant concentration preceding the pandemic to measure chronic TRAP concentrations and calculate all hours during that time period for which the census tract lies downwind of the nearest highway. This 10-year period is slightly shorter than the average tenure time of residents in NYC census tracts according to the 2018 ACS, and there is no difference in median tenure time across our upwind and downwind census tracts (S3 Table in S1 File), so household movement across tracts over time should not bias our coefficient estimates. Specifically, we construct the instrumental variable $\hat{AQ}$, the exogenous component of air quality (AQ), by modelling AQ as a linear combination of wind-related variables and other exogenous observables.

There are three AQ measures in our study: PM_2.5_, NO, and NO_2_. The health effects of these pollutants have been well studied and reviewed by the United States’ Environmental Protection Agency [38, 39]. Furthermore, both PM and NO_x_ are established surrogate pollutants used to evaluate overall exposure to TRAP and its impact on health [40]. No one pollutant is a sufficient surrogate, so we chose both measures of NO_x_ and PM_2.5_ as surrogates for overall TRAP exposure in this analysis.

In the second stage, we estimate the effect of this exogenous portion of pollution $\hat{AQ}$ on COVID-19 outcomes, as measured by mortality and hospitalizations recorded at the census-tract level between February 29 and August 30, 2020. This period is prior to vaccine availability, which could confound the relationship, as individuals at high-risk were often prioritized for vaccines and uptake has been shown to vary by observable individual characteristics [41]. Our dense monitoring network and tract-level COVID-19 data disaggregated by age and race-ethnicity represent improvements relative to existing studies that have relied on county-level data.

### First-stage regression

We first predict PM_2.5_, NO_2_ and NO concentrations in each census tract as linear functions of the percent of time that the tract spends downwind of a highway at each distance:

$${AQ}_{i}=\eta +\beta {Downwind}_{i}+\mu {HighwayDistance}_{i}+PUMA_{i}+Station_{i}+\epsilon_{i}$$ (Eq 1)

*Downwind_i_* is the percent of time that census tract *i* is downwind of any highway segment that is located within 0.5 kilometers (km) across a 10-year period (2009–2018). The model also controls for distance to the closest highway, *HighwayDistance_i_*, dummy variables for Public Use Microdata Areas (PUMAs), *PUMA_i_*, and dummy variables for the nearest weather station, *Station_i_*.

The key assumptions necessary for instrument validity are (1) the instruments are correlated with the pollutant concentrations (relevancy), and (2) the instruments must not be related to COVID-19 disease intensity except through their relationships with pollutant concentrations (exclusion restriction). The relevancy assumption is met–the downwind variable is significantly associated with increased pollution concentration at distances less than 0.5 km (see S2 Table in S1 File). Focusing on the Citywide models, we find that a tract within 0.5 km of a highway and downwind 100% of the time would have increased average ambient concentrations by 0.32 μg/m3 of PM_2.5_, 0.70 ppb of NO_2_ and 1.41 ppb of NO, relative to a tract that is downwind 0% of the time. We report the weak instrument Wald F-stats from the first stage and reject the null hypothesis that the instrument is irrelevant.

As for the exclusion restriction assumption, while exposure to poor air quality as a result of living near a highway is likely endogenous with factors related to COVID-19 disease intensity (e.g., income, healthcare access, etc.), we assume that, conditional on living near a highway and in the same neighborhood, our instrument only affects COVID-19 outcomes through its effect on pollution concentration. This assumption is reasonable because the pollutants of interest are generally not detectable via sight or smell at concentrations in NYC, and differences of the magnitudes our coefficients report would clearly not be detectable by human senses.

To further ensure that our instrument for ambient air quality is unrelated to a set of observable characteristics, **X**_i_, that might determine health outcomes, we run placebo regressions of the following form, where *x_i_* denotes each element in the observable characteristics set **X**_i_:

$$x_{i}=\eta +\beta {Downwind}_{i}+\mu {HighwayDistance}_{i}+PUMA_{i}+Station_{i}+\epsilon_{i}$$ (Eq 2)

The results of these regressions are reported in S3 Table in S1 File, which presents the coefficients and standard errors from regressing each of our included demographic characteristics on our air quality instrument. For our sample of interest, the set of census tracts that lie between 0.05 and 0.5 km from the nearest highway, the coefficient on the percentage of Black residents is marginally significant at the 10% level, which we would expect to occur by chance given our set of 12 control variables. In the Outer Boroughs sample, we observe a marginally significant (at the 10% level) increase in the number of people living in downwind census tracts relative to upwind census tracts. This correlation would bias our estimate upward, making our estimated null effects even more robust. These results give us great confidence that our instrument allows us to identify the causal effect of ambient air quality on COVID-19 outcomes in our analysis.

We next test the assumption that populations in our main sample that spend relatively more time downwind of highways are similar to those that spend less time downwind. To do so, we conduct balance checks comparing variables constructed from ACS data representing tract level socioeconomic and demographic characteristics. A tract is considered “Downwind” if the amount of time the tract spends downwind of a highway is greater than the average amount of time tracts the same distance from a highway spends downwind and is considered “Upwind” otherwise. We show that no normalized differences between Upwind and Downwind tracts are greater than 0.25, supporting our assumption that, conditional on living in a tract near a highway, there are not significant socioeconomic or demographic differences between tracts based on the amount of time they spend downwind of highways (S4 Table in S1 File).

While there are many reasons for residential sorting to occur in NYC, the above results suggest that it is unlikely that individuals are sorting into tracts based on the amount of time the tract spends downwind of a highway. For this reason, we assume that our instruments are exogenous, and that the exclusion restriction is met.

### Second-stage regression

Using our quasi-experimental design to capture the variation in ambient pollutant concentration that is uncorrelated with demographic characteristics, our second-stage regressions estimate the causal impact of a change in ambient pollutant concentration on two different measures of the intensity of COVID-19 disease: deaths and hospitalizations. Our identification strategy compares tracts within the same PUMA that lie within the same distance of the highway that spent different amounts of time downwind from 2009–2018. Our key specification is:

$$\log\left(Y_{i}\right)=\alpha_{0}+\alpha_{1}\hat{AQ_{i}}+\alpha_{2}{HighwayDistance}_{i}+PUMA_{i}+Station_{i}+\nu_{i}$$ (Eq 3)

Y_i_ are counts of deaths and hospitalizations. We use log-transformed counts and add one to the count variables to adjust for tracts with zero deaths or hospitalizations. $\hat{AQ_{i}}$ is the instrumented measure of ambient concentration for PM_2.5_, NO_2_, and NO predicted from Eq (1). We include weather station and PUMA dummy variables in the second stage as well. The key parameter of interest is α_1_. Please see the S1 File for more details on our data construction, methods, results, and discussion of our robustness checks.

## Results

**Fig 3:**
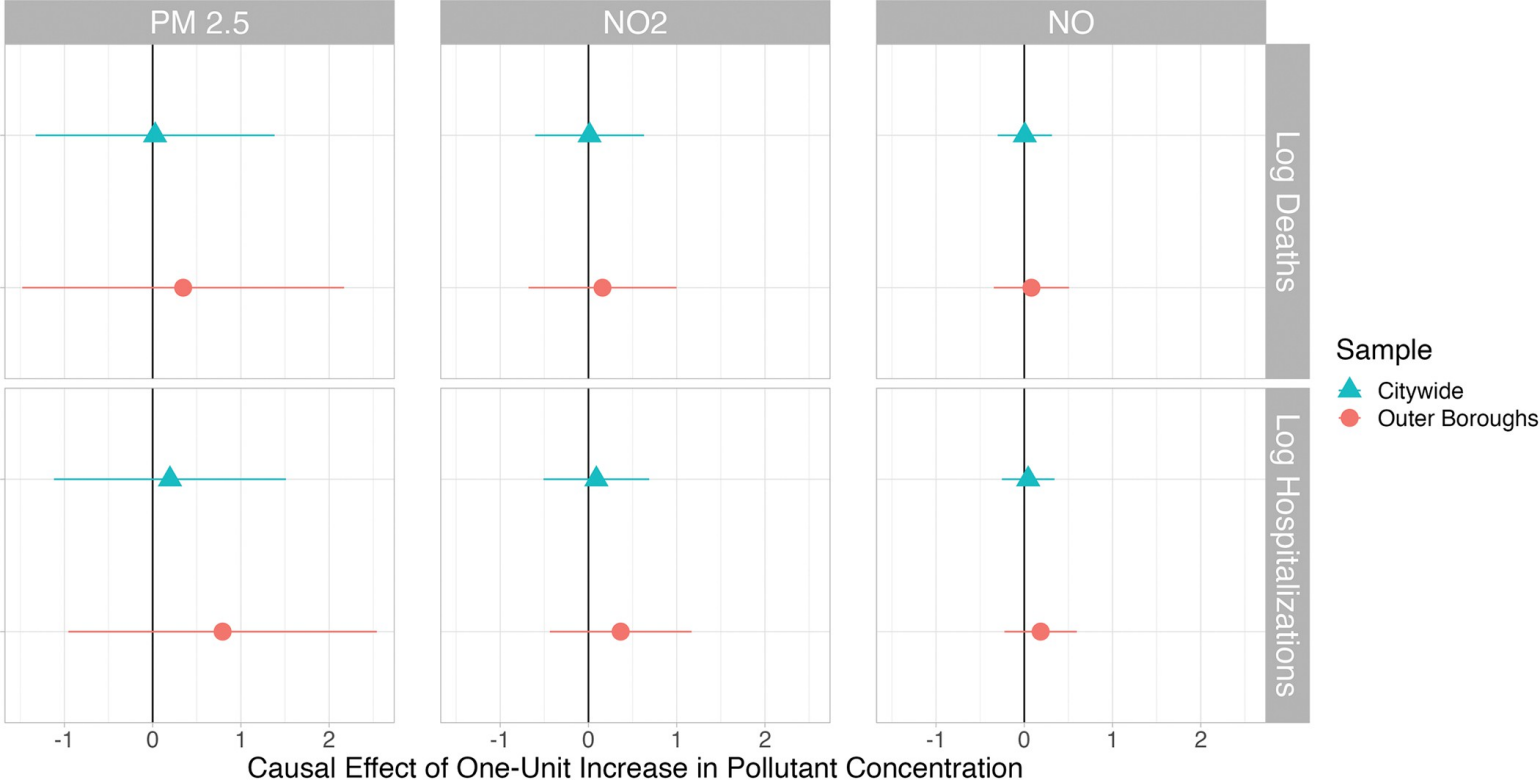
This figure presents the estimated coefficients and associated 95% confidence intervals on our instrumented measure of ambient pollutant concentration from the second stage of our instrumental variable log-linear models across our two geographic samples for all tracts that lie between 0.05km and 0.5km of the nearest highway.

Fig 3 presents the causal effects of chronic exposure to three TRAPs on COVID-19 deaths (row 1) and hospitalizations (row 2). The figure panels illustrate results for two sets of observations: Citywide, which uses all tracts in NYC that lie between 0.05km and 0.5km of the nearest highway, and Outer Boroughs, which includes all such tracts in the Bronx, Brooklyn, Queens, Staten Island, and above 110th street in Manhattan. The point estimate of the causal impact of increased chronic pollutant concentration is near zero and statistically insignificant for each of our considered pollutants, whether we are considering COVID-19 deaths or hospitalizations. We see a much tighter confidence interval for NO_2_ and NO, the pollutants that are more directly related to traffic. Our findings are also robust to employment of an IV Poisson model and the addition of demographic control variables. (See S5, S6, S12, S13 Tables in S1 File).

**Fig 4:**
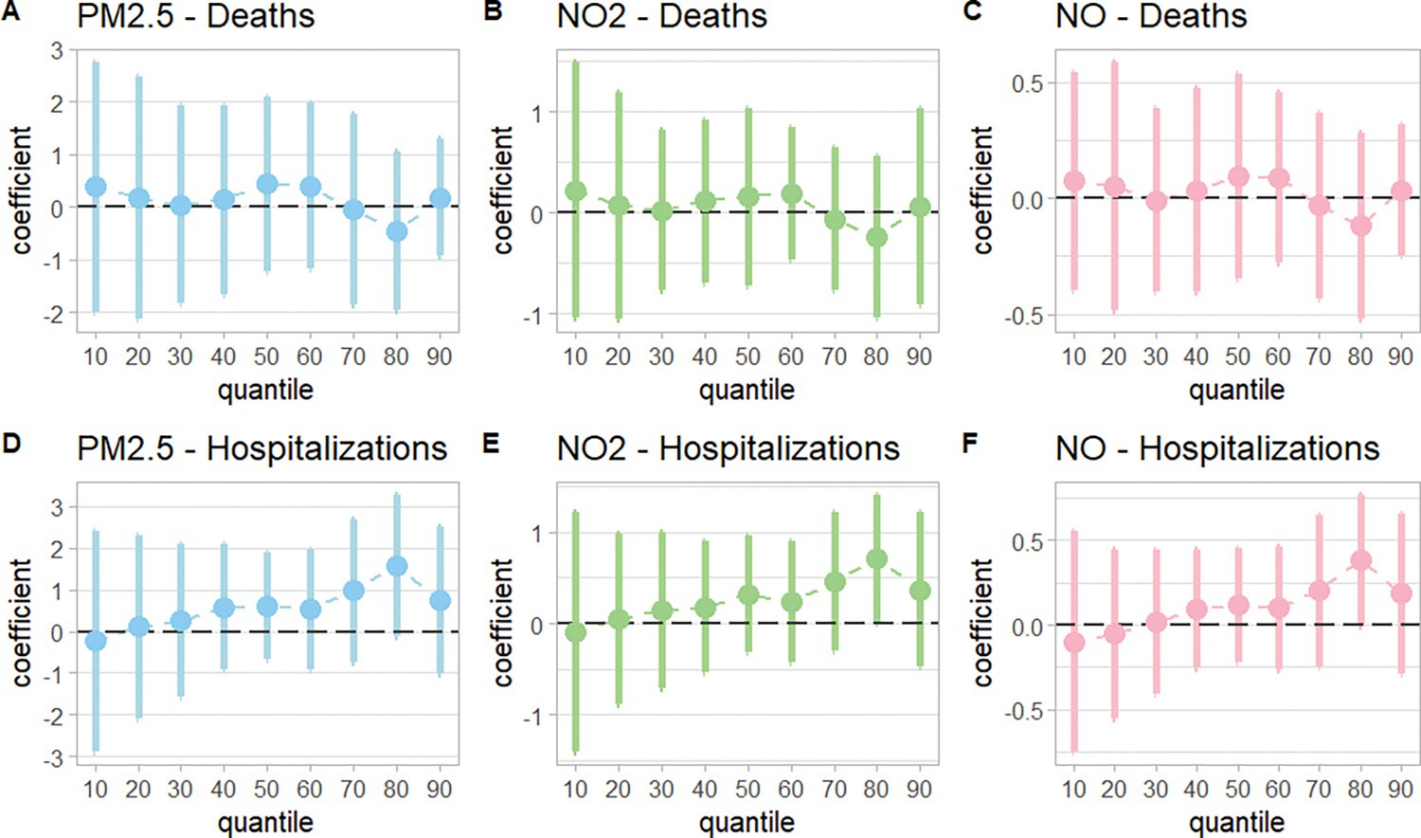
Quantile regression results for our focal COVID-19 outcomes in the sample of NYC census tracts that lies between 0.05km and 0.5km of the nearest highway.

We also perform quantile IV models to explore how the effects of air quality operate across the distribution of tracts by COVID-19 deaths. Fig 4 shows the quantile regression estimates for our Citywide sample, with point estimates that are also consistently near zero across tracts based on their position in the distribution of COVID-19 deaths.

While the point estimates are still statistically insignificant, we see evidence that the effect on COVID-19 hospitalizations is more pronounced in the tracts with a higher number of hospitalizations, which are located in the Washington Heights neighborhood of northern Manhattan. These tracts have three times the recorded population of an average tract, with Hispanic individuals representing roughly 80% of the total population. This finding is consistent with those from our heterogeneity analyses: when we interact our instrumented measure of pollution with age and race-ethnicity groups (see S9 and S10 Tables in S1 File), we find some evidence that TRAPs increase hospitalizations and deaths for Hispanic individuals. These results are not significant at the 95% threshold when we adjust our threshold p-values to account for multiple hypothesis testing, though they may warrant further investigation. For all other age and race-ethnicity groups, we do not detect meaningful heterogeneity in the effect of TRAPs on COVID-19 deaths or hospitalizations.

**Fig 5:**
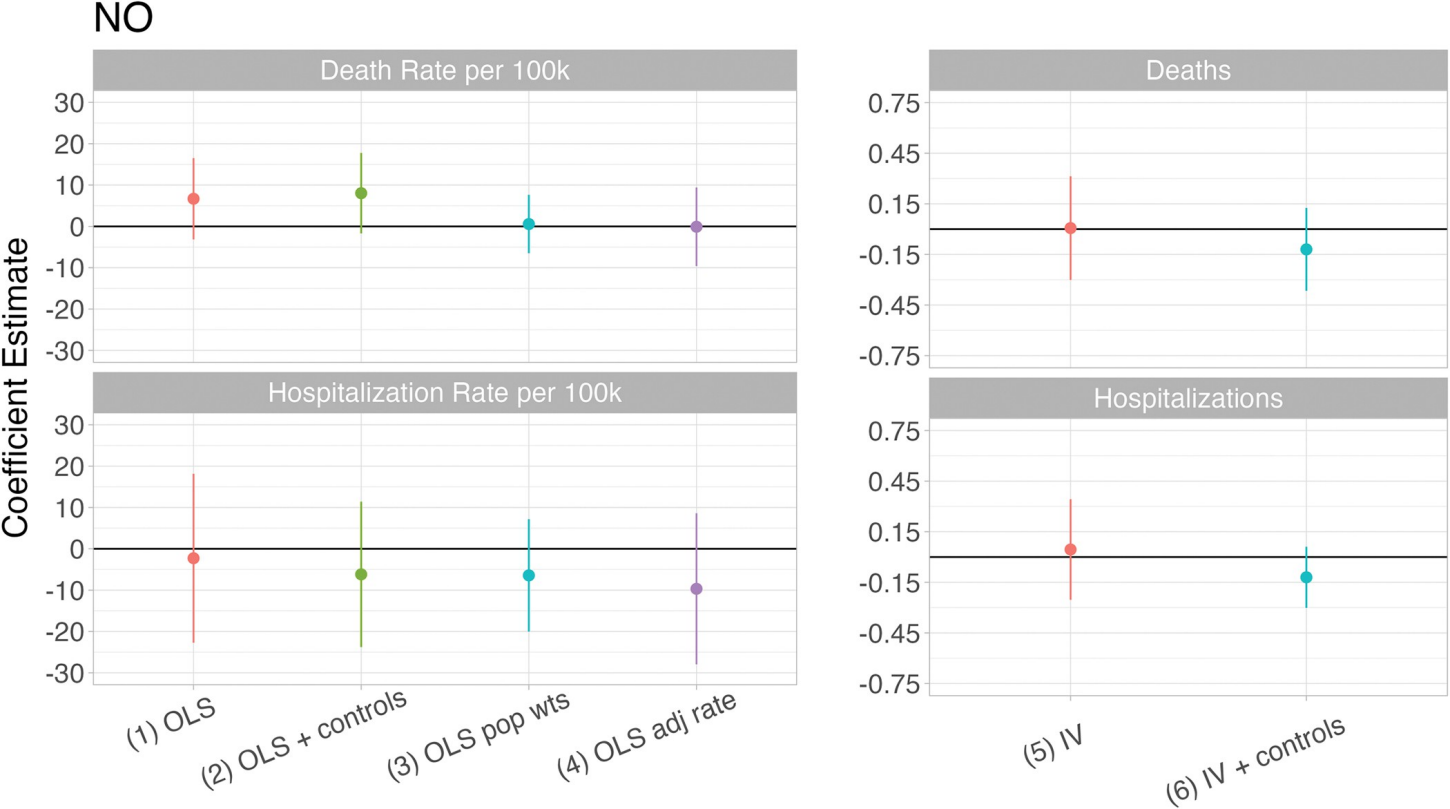
This figure presents coefficient estimates for various models exploring the impact of increased ambient NO concentration on COVID-19 deaths and hospitalizations. OLS models (left panel) do not capture the causal effect of air pollution due to their endogeneity. These rate-based models are also impacted by measurement error in the dependent variable that is correlated with unobservables that determine COVID-19 outcomes. These rate-based models are still impacted by measurement error in the dependent variable when covariates are included, while the inclusion of these covariates can bias estimation by conditioning on post-treatment variables. The IV models (right panel) capture the causal effect of air pollution on COVID-19 deaths, through both direct and indirect channels in models without covariates.

Fig 5 presents a series of replications of existing studies using our data and shows coefficient estimates for various models exploring the impact of increased ambient NO concentration on COVID-19 deaths and hospitalizations. The left panels are estimated with ordinary least squares (OLS) models of rate-based outcomes and the right panels are estimated with count-based IV regressions, our preferred models. With an OLS model that controls for a representative set of demographic variables, we find that increasing NO concentration by 1 ppb leads to an imprecisely estimated additional 8 deaths per 100,000 in our sample tracts (model 2). However, this positive significant effect diminishes when we account for population weights in running the OLS model (model 3). Finally, when we incorporate the Safegraph mobility data to adjust for the mortality rate (model 4), we find a null effect, which is qualitatively consistent with our main finding, as shown in the right panels (model 5).

## Discussion

Empirical researchers studying the effects of air quality must confront the shortcomings of working with observational data. We use the recent SARS-CoV-2 global pandemic to highlight three issues in particular: 1) Non-random exposure to pollution, 2) coarse measurement of pollutant exposure, and 3) endogenous defensive behaviors. We show that a number of observational studies that generally report large correlations between ambient pollutant concentrations and COVID-19 outcomes fail to address most, if not all, of these challenges, meaning that the conclusions of these papers might offer misleading advice to policymakers.

To illustrate how these theoretical challenges to causal inference might bias estimated correlations, we take advantage of unusually spatially-dense air quality monitoring in NYC, and a quasi-experimental approach to identify the causal relationship between chronic ambient concentrations of several TRAPs and the intensity of COVID-19 disease. We also highlight how defensive behaviors bias rate-based measures of COVID-19 severity. The results of our instrumental variables analysis show that increases in the average chronic concentration of three TRAPs do not cause the substantial impacts on COVID-19 outcomes in NYC that have been reported in observational studies. The differences between our methodological approaches and results relative to the existing literature on air quality and COVID-19 might be interpreted as evidence that observational studies can generate biased estimates of pollution impacts that could be misinterpreted by policymakers. In fact, the only study that does not use an observational approach [7], reports smaller effects than most of the observational studies, though its findings are still susceptible to the other two identification issues that we address in this paper.

The goal of this paper is to highlight the challenges of identifying causal effects of air quality on human health, emphasizing the challenge of systematic measurement error, which has been seriously understudied relative to the issue of endogenous ambient air quality. To do so, we have relied on a uniquely spatially-dense network of monitors in a relatively small geographic area. Our results are not consistent with the large, statistically-significant correlations reported in the observational studies included in Table 1. That said, our small study area limits variation in air quality conditions, and our focus on the causal effect of chronic ambient conditions makes it challenging to detect small effects. With our dataset, we can say that a 1 ppb increase in the concentration of NO could cause no more than 2.32 additional COVID-19 deaths at the census tract level (an increase of 32% relative to the average of 7.25 tract-level deaths in our main sample). We also note that our ability to identify causal effects in this context is limited by the metrics available at the census tract level regarding COVID-19 outcomes. While we focus on measures related to the extensive margin (e.g., counts of deaths and hospitalizations), future study that is able to acquire patient-level data might find additional impacts by considering metrics that reflect impacts on the intensive margin (e.g., ventilator use for hospitalized patients in the context of COVID-19).

That we do not detect a causal link between chronic ambient air pollutant concentrations and COVID-19 outcomes in NYC does not mean that such a causal impact might not exist in other contexts. Our estimated effects are observed at chronic ambient pollutant concentrations that fall below the NAAQS thresholds for PM_2.5_ (three-year annual average of 12 μg/m^3^ for the primary standard) and NO_2_ (annual average of 53 ppb). Furthermore, while we have been careful in our analysis to identify the exogenous portion of variation in ambient TRAP concentrations, the unique aspects of NYC might limit the generalizability of our findings.

We emphasize that our estimate of air quality might not fully capture the 10-year cumulative exposure to air pollution. Rather, it only captures the average ambient air pollutant concentration. This limitation relates to the challenge of proxying for a stimulus variable with ambient concentration measures, as the ability to moderate exposure to air pollution via defensive behaviors may differ across individuals. We have shown these defensive behaviors to be very important in the context of COVID-19, which is just one of the respiratory ailments through which air-pollutant exposure might lead to premature death and reduced quality of life. This measurement is a challenge for all existing work that has explored the health effects of air quality and their implications for behavior, whether through observational or quasi-experimental study. While researchers can take steps on their own to address the challenge of endogenous air quality (or measurement error in the dependent variable in the case of future pandemics), addressing the issue of mismeasured pollutant exposure most likely involves expanded investment in air-quality monitoring networks around the world.

Despite these caveats, our results suggest that air quality may not be as significant a determinant of susceptibility to SARS-COV-2 as correlated socio-economic characteristics. Previous research that has emphasized such a link may be confounding the impacts of air quality with other determinants of poor health or disease exposure.

By highlighting the challenges facing studies exploring the impacts of air quality on health and demonstrating methods to address these challenges, we hope to improve the policy-relevance of information generated by economic and public-health research in this area. While strands of the literature have acknowledged that air quality does not vary randomly across space, much less attention has been paid to the impacts of systematic measurement error related to measures of exposure to air pollutants. Our study shows that this issue is of critical importance in accurately estimating causal effects. Adopting measures to address the three key challenges to causal identification should lead to research results that can improve the efficacy of public-health interventions related to air quality.

We hope that our presentation of these key challenges makes it easier for public-health officials and policymakers in general to distinguish between mere correlations and causal relationships, which should be used for policy development.

## Supporting information

S1 File (PDF)
