# Supplementary material for: Observational studies generate misleading results about the health effects of air pollution: Evidence from chronic air pollution and COVID-19 outcomes
Source: PLoS One. 2024 Jan 2;19(1):e0296154. doi: 10.1371/journal.pone.0296154 (PMC10760733; doi:10.1371/journal.pone.0296154)
Supplement: S1 File — (PDF) [file pone.0296154.s001.pdf]

Supplementary Materials for Observational studies generate misleading results about the health effects of air pollution: evidence from chronic air pollution and COVID-19 outcomes

Marc N. Conte[*], Matthew Gordon[†], Nicole A. Swartwood[‡], Rachel Wilwerding[§], and Chu A.(Alex) Yu[¶]

September 13, 2023

[*]Department of Economics, Fordham University, corresponding author: marc.conte@fordham.edu
[†]Department of Environmental Studies, Yale University
[‡]T.H. Chan School of Public Health, Harvard University
[§]Department of Economics, Fordham University
[¶]Department of Economics, Wake Forest University

Contents

1 Data **3**

2 Methods **7**

2.1 Constructing Wind-Related Instruments 8

2.2 First-Stage Regression 12

2.3 Second-Stage Regression 18

2.3.1 Log-linear model 18

2.3.2 Poisson model 18

3 Results and Discussions **19**

3.1 Main Specifications 19

3.2 Challenges to Causal Identification 22

3.3 Age and Race-Ethnicity Regressions 26

3.4 Count-Based vs Rate-Based Measures 29

3.5 Demographic Controls 32

3.6 Additional Robustness Checks 34

4 Supplement References **37**

5 Software References **37**

Background

The recent economic literature on the health effects of acute air-quality conditions includes efforts to accommodate the endogeneity of air quality using weather-related variables. Weather-related variables provide identifying variation in pollution that is unlikely to be correlated with unobserved determinants of health outcomes (27-29).

Conducting our study in a relatively small spatial area allows us to avoid issues that challenge cross-sectional observational studies conducted over larger regions (e.g., national studies), such as correlation between air pollutant concentrations and employment opportunities. In addition, it allows us to take advantage of a dense local pollution monitoring network.

We use an instrumental-variable approach to identify the causal impact of chronic air quality conditions on the intensity of COVID-19 infection in New York City, using the fraction of time spent downwind of highways at different distances from the census tract as instruments for chronic air quality conditions. Our strategy allows us to compare tracts in the same neighborhood that lie the same distance from the highway, but experience different amounts of time downwind of the highway.

1 Data

Our analysis is based on data from several different sources. The census-tract level data on COVID-19 case, hospitalization (hospitalizations within $\pm$ 14 days of the diagnosis date), and death (confirmed and probable) counts comes from the New York City Department of Health and Mental Hygiene, covering all census tracts in New York City (NYC) from February 29, 2020 to August 30, 2020. The data set includes 18,856 deaths and 48,427 hospitalizations stratified by census tract, race, and age, but does not include deaths or hospitalizations in institutionalized settings (prisons and nursing homes).

We use the New York City Community Air Survey (NYCCAS) monitor readings to estimate ambient concentrations of traffic related air pollution (TRAP), focusing on $PM_{2.5}$, NO_2, and NO. We calculate the 10-year average concentration of each pollutant from 2009-2018, which is slightly shorter than the average of the median years that individuals have lived in their current residents across the city (11.7). There were between 66 and 110

PM2.5 Monitoring Network

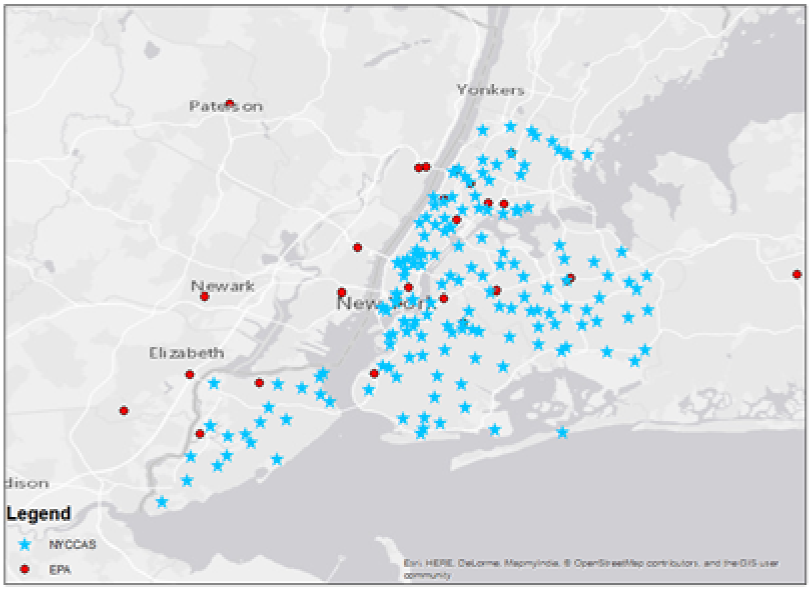

S1 Figure: $PM_{2.5}$ Monitoring Network in NYC

NYCCAS monitors active each year between 2009 and 2018, as compared to the 12 EPA monitors in the city, allowing us to avoid several issues associated with the sparse and strategically-sited EPA monitoring network (22,23) and satellite-derived air-quality data that are downward biased (24), calibrated to ground-based networks (25), and too coarse to detect our observed relationships given the rapid decay of ambient concentrations of TRAP with increasing highway distance. Figure S1 plots the $PM_{2.5}$ monitoring network for both NYCCAS and EPA monitors.

We take bi-weekly pollution concentrations measured at each monitoring site and calculate 10-year average pollutant concentration for each census tract centroid in NYC, using squared inverse distance weighted (IDW) averages. Our results are also robust to proxying for exposure with average ambient pollution measured from the closest monitor (see Table S13, panel B).

We collect hourly wind direction data from the National Centers for Environmental

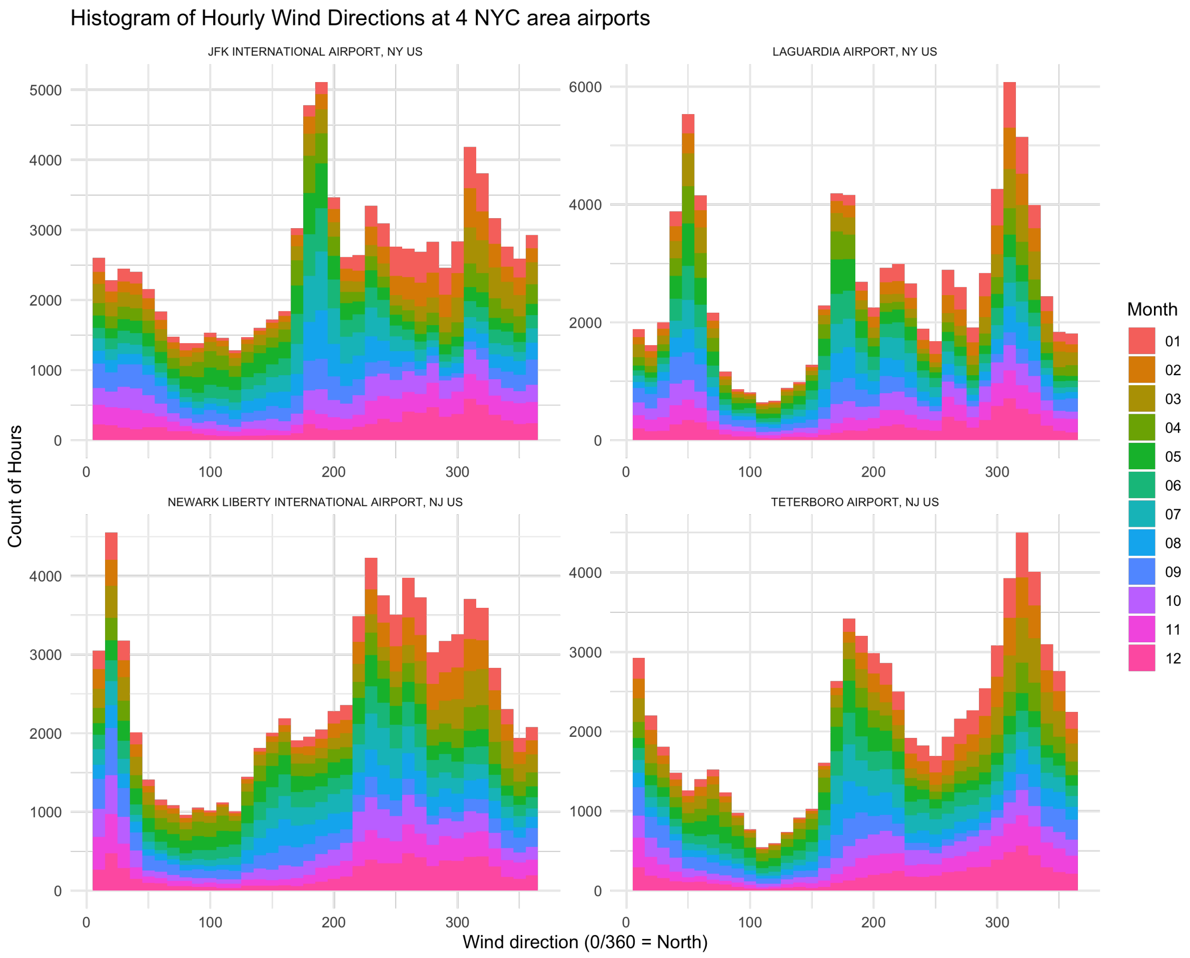

S2 Figure: Histogram of Hourly Wind Directions at 4 NYC-area Airports

Information (NCEI) Integrated Surface Database (ISD). We use this data to develop a count of the hours during our study period that a census tract is downwind of the nearest highway to calculate the fraction of time a census tract is downwind of a highway over a 10-year period. When constructing our measures from the closest monitor to each census tract, we rely on data from JFK, Newark, La Guardia, and Teterboro airports. Histograms of hourly wind directions are shown in Figure S2.

We incorporate cell phone mobility data from Safegraph to examine avoidance behavior. Safegraph collects location data from 45 million mobile devices and provides aggregated statistics at the census block group level on the amount of time that mobile devices are in the home, outside of the home, and engaged in work behavior, as well as

distance traveled. We aggregate this data to the census tract level to match with our outcome variables.

Our socioeconomic and demographic variables come from the 2018 5-year American Community Survey (ACS) and are used in some of our robustness checks. In our main specifications, we use tracts with more than 500 people. Our results are robust to using a threshold population of 100 as well (see Table S13, panel A). The average tract population for our main sample is 4,251 individuals. 68 out of 2,165 tracts have fewer than 500 people and 55 have fewer than 100 people. Most of these tracts are parks or non-residential areas. Summary statistics for all tracts in our main specification are presented in Table S1.

To generate exogenous measures of our focal TRAP components, we use information about each tract's orientation to the highway network in NYC. GIS data on NYC's highways comes from TIGER/Line Shapefiles (2015) for primary and secondary roads in NYC. Our interest is in a census tract's position relative to highway segments in NYC. To obtain accurate measurements of the distance and direction from each census tract centroid to surrounding highways, we divide the city into a grid with .01 by .01 degree cells (approximately 1.11 km on each side). Any lengths of highways within each grid cell are considered a 'highway segment'.

S1 Table: Summary Statistics

A. Summary Statistics: Citywide

	N	Mean	St. Dev.	Min	Pctl(25)	Pctl(75)	Max
Tract Population	842	4,245.78	2,345.64	537	2,455.8	5,417.2	16,684
Downwind < 0.5km	842	0.22	0.12	0.04	0.14	0.27	0.83
Highway Distance (m)	842	0.27	0.12	0.05	0.17	0.36	0.50
$PM_{2.5}$ (ug/m3)	842	9.49	0.76	8.02	8.89	9.93	12.24
NO_2 (ppb)	842	21.86	1.70	16.84	20.73	22.60	27.84
NO (*ppb*)	842	21.71	2.54	15.55	19.92	23.01	31.30
Per Capita Income	840	35,108.75	26,142.25	2,634.11	19,474.66	39,315.36	195,124.40
Change in 'Home' Devices	841	−0.14	0.16	−0.82	−0.21	−0.05	0.43
COVID-19 Deaths	842	9.57	7.54	0	4	13	50
COVID-19 Hospitalizations	842	25.14	19.25	0	12	35	166
COVID-19 Cases	842	104.81	68.27	7	54	138.8	491

B. Summary Statistics: Outer Boroughs

Statistic	N	Mean	St. Dev.	Min	Pctl(25)	Pctl(75)	Max
Tract Population	751	3971.76	2097.84	537	2360	5146	15456
Downwind < 0.5 km	751	0.22	0.12	0.04	0.14	0.27	0.83
Highway Distance (m)	751	267.35	115.88	50.64	172.80	357.74	499.38
$PM_{2.5}$ (ug/m3)	751	9.33	0.56	8.02	8.86	9.77	10.85
NO_2 (ppb)	751	21.48	1.24	16.84	20.66	22.33	24.91
NO (*ppb*)	751	21.22	1.91	15.55	19.86	22.37	27.57
Per Capita Income	749	29264.57	15054.33	2634.11	18896.48	35192.28	111963.00
Change in 'Home' Devices	751	-0.07	0.14	-0.50	-0.15	-0.01	0.86
COVID-19 Deaths	751	9.77	7.69	0	4	13	50
COVID-19 Hospitalizations	751	25.62	19.76	0	12	35	166
COVID-19 Cases	751	106.99	70.17	7	54	143	491

Notes: This table shows summary statistics for our main sample: census tracts 0.05 to 0.5 km of a highway with at least 500 residents. Summary statistics are presented for the percent of time tracts in the sample spent downwind of a highway 2008-2018, distance from tract centroids to the nearest highway (m), 10-year average concentrations for each of our pollutants of interest, tract populations, per capita incomes, percentage changes in 'home' devices, and measures of COVID-19 infection intensity in terms of number of cases, hospitalizations, and deaths at the tract level. Panel A displays these summary statistics for the entire city, and Panel B shows statistics for the Outer Borough sample.

2 Methods

It is well documented that air pollution is correlated with many socioeconomic variables that may influence COVID-19 outcomes. Even a rich set of control variables may result in

biased estimates of the effect of air quality if some of the covariates have multi-directional causality with air quality, or if there are interaction effects between variables. To overcome this issue, we use an instrumental variables approach that relies on variations in pollution resulting from wind direction relative to nearby highways to identify the causal effects of chronic ambient air pollution on COVID-19 disease intensity. A key assumption underlying this method is that wind direction is exogenous, or uncorrelated with individual or community characteristics that are correlated with COVID-19 outcomes. In other words, wind direction only effects COVID-19 outcomes through its effect on air quality.

We use a two stage least squares (2SLS) technique to isolate the exogenous component of air quality (AQ). As the name suggests, the estimation can be decomposed into two stages. In the first stage, we construct the instrumental variable $\widehat{AQ}$ by modelling AQ as a linear combination of wind-related variables and other exogenous observables. $\widehat{AQ}$ can be considered the 'exogenous portion' of air quality. In the second stage, we regress COVID-19 outcomes on the exogenous $\widehat{AQ}$ and the same set of exogenous observables.

Section 2.1 describes how wind-related variables are constructed, section 2.2 provides the details on the first-stage regression, and section 2.3 provides the details on the second-stage regression.

2.1 Constructing Wind-Related Instruments

Ambient pollutant concentrations in a census tract depend on both distance and direction to nearby highways. So, for each census tract m, we first calculate the angle θ_{mn} between the tract centroid and the nearest point on each highway segment n within 5km of the tract centroid, as well as the distance d_{mn} to the nearest point on each highway segment. A tract is considered downwind at a certain distance in any given hour if there exists at least one θ_{mn} such that the absolute difference between θ_{mn} and the wind direction measured at the nearest NCEI weather station is less than 45° and d_{mn} lies within a certain range. We focus on near highway tracts with $d_{mn} < 0.5$km. We then calculate the fraction of hours during our 10-year period that each tract is downwind at each of these respective distance ranges. Next, we construct a set of corresponding instruments, denoted "$downwind_k$", as the percent of time downwind of any highway segment that is located in range "k" km across a 10-year period.

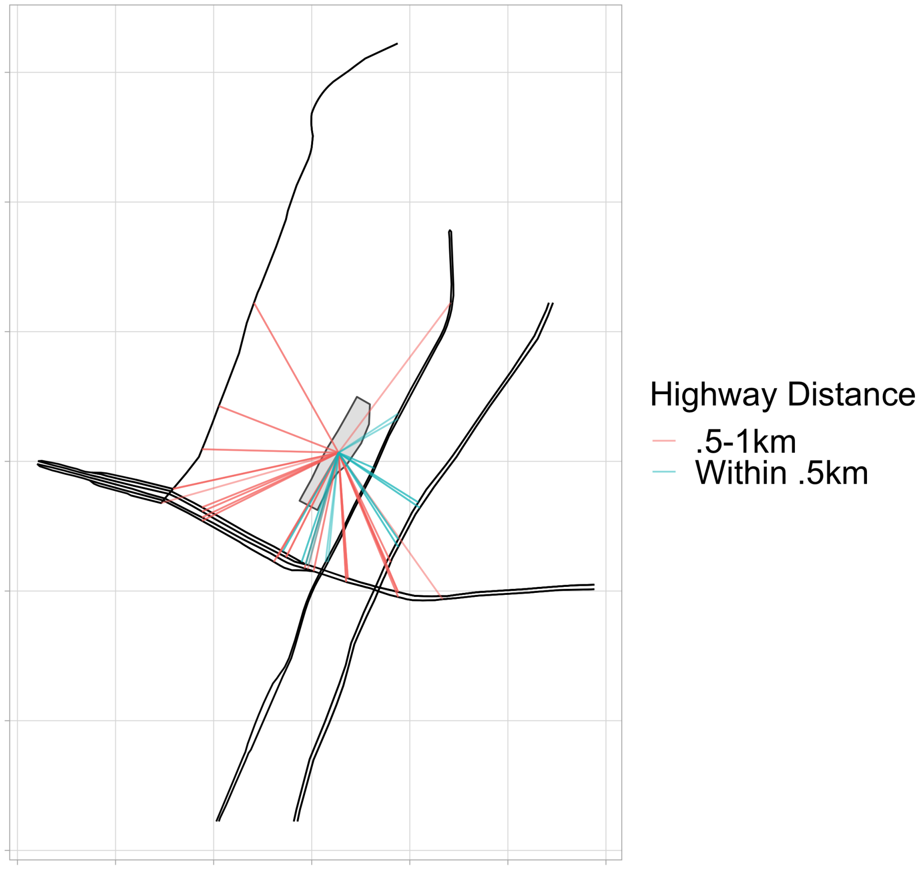

S3 Figure: Calculating Distance and Orientation from a Census Tract to Nearby Highways

Note: Black lines show highways, red and blue rays show distance from census tract centroid to closest point on each highway segment. Blue lines connect the tract to segments between 0 and 0.5km. Red lines go to segments between 0.5 and 1km.

Figure S3 illustrates this for an example census tract. The lines go from an example census tract centroid to the nearest point on each highway segment, with colors corresponding to distance groupings. This tract would be downwind at 1km for an hour where the wind blows from the North or West for example, and downwind at 0.5km for an hour where the wind blows from the South or East. Note, the tract could be downwind at multiple distances simultaneously.

It is critical to note that tracts that straddle both sides of a highway are subject to significant measurement error, as these tracts could be recorded as downwind (or not) on a day when a significant fraction of their population is actually upwind (or downwind). This measurement error is crucial, because tracts that are very close to highways are likely to differ in several respects compared to other tracts. For this reason, in our main sample, we drop tracts with centroids that are very close to a highway (within 0.05km), following Anderson (25). For reference, the median area of an NYC census tract is slightly less than $2km^2$, or, if a square is assumed, 1.4km on a side. Figure S4 shows the fraction of time downwind at 0.5km, corresponding to our main specification. Although there are geographic patterns in these variables, our identification relies on variation *within neighborhoods* (outlined in grey). Therefore, we essentially compare tracts that are on different sides of the same highway.

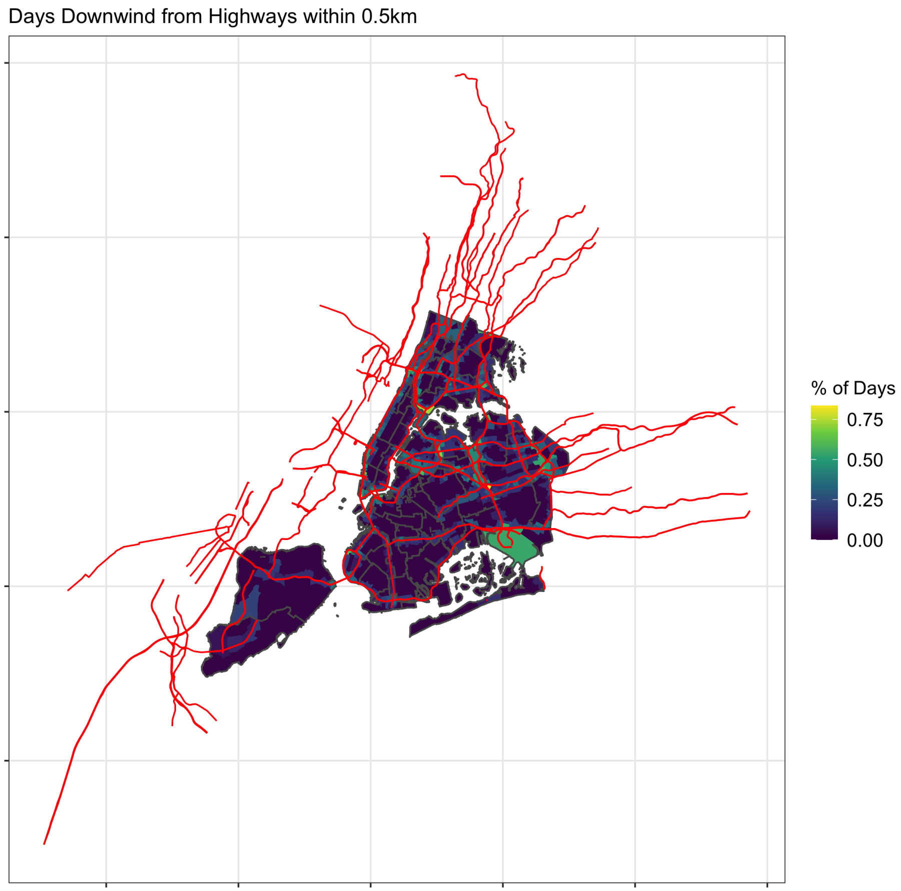

S4 Figure: Fraction of time downwind for tracts within 0.5km of a highway.

Note: Highways in red, PUMAs outlined in grey.

2.2 First-Stage Regression

In the first stage, we predict $PM_{2.5}$, NO_2 and NO as linear functions of the percent of time downwind of a highway at each distance:

$$AQ_i = \eta + \beta Downwind_i + \mu HighwayDistance_i + PUMA_i + Station_i + \varepsilon_i \quad (1)$$

$Downwind_i$ is the percent of time that census tract i is downwind of any highway segment that is located within 0.5 km across a 10 year period. The model also controls for distance to the closest highway, $HighwayDistance$, dummy variables for Public Use Microdata Areas (PUMAs), and dummy variables for the nearest weather station.

The key assumptions necessary for instrumental validity are (1) instruments are correlated with the pollutant concentrations (relevancy), and (2) instruments must not be related to COVID-19 disease intensity except through their relationships with pollutant concentrations (exclusion restriction). The relevancy assumption is met - as shown in Table S2, the downwind variables are significantly associated with increased pollution concentration at distances less than 0.5 km. Focusing on the Citywide models, models (1)-(3) suggest that a tract within 0.5 km of a highway and downwind 100% of the time would have increased average ambient concentrations by 0.32 $\mu g/m^3$ of $PM_{2.5}$, 0.70 ppb of NO_2 and 1.41 ppb of NO, relative to a tract that is downwind 0% of the time. Table S2 models (4)-(6) show the first stage regressions using the Outer Borough sample and models (7)-(9) using all NYC tracts that are within 1 km of the nearest highway. We report the weak instrument Wald F-stats from the first stage and reject the null hypothesis that the instruments are irrelevant. In our age-by-race stratified regressions, we have a weak instrument problem (Wald F stats < 10) for the results in our Asian over-65 subsample, and thus we have less confidence in those estimates (see section 3.3, table S10).

Table S2: Results from First Stage Regressions

	PM2.5	NO2	NO	PM2.5	NO2	NO	PM2.5	NO2	NO
	(1)	(2)	(3)	(4)	(5)	(6)	(7)	(8)	(9)
Downwind < 0.5km	0.32***	0.70***	1.41***	0.26***	0.56***	1.10***			
	(0.06)	(0.14)	(0.27)	(0.06)	(0.13)	(0.27)			
Downwind < 1km							0.21***	0.44***	0.86***
							(0.04)	(0.08)	(0.16)
Wald F-stats	22.86	21.14	22.49	20.01	19.35	18.64	20.83	18.42	19.13
Geography	Citywide	Citywide	Citywide	Outer Borough	Outer Borough	Outer Borough	Citywide	Citywide	Citywide
N	842	842	842	751	751	751	1,407	1,407	1,407

Notes: This table shows results from the first-stage regression analysis of our instrument. The dependent variables are the inverse-distance weighted chronic ambient pollution concentrations estimated for centroids of the census tracts between 0.05 km-0.5 km and 0.05 km-1 km of a highway with populations greater than 500 people. Downwind < 0.5 km refers to the sample of tracts downwind of a highway within 45°of the incoming wind direction between 0.05 and 0.5 km away from the closest highway, and Downwind < 1 km is the sample of tracts within 45°of the incoming wind direction between 0.05 and 1 km of the nearest highway. Each model includes the following additional controls: distance to the nearest highway, and indicators for PUMA and the nearest weather station. Robust standard errors are reported in parentheses. ***, **, and * indicate significance at the 1, 5, and 10 percent levels, respectively.

As for the exclusion restriction assumption, while exposure to poor air quality as a result of living near a highway is likely endogenous with factors related to COVID-19 disease intensity (e.g., income, health care access, etc.), we assume that, conditional on living near a highway and in the same neighborhood, our instruments only affect COVID-19 outcomes through their effect on pollution concentration. This is a reasonable assumption because the pollutants of interest are generally not detectable via sight or smell at concentrations in NYC, and differences of the magnitudes our coefficients report would clearly not be detectable.

To further ensure that our instrument for ambient air quality is unrelated to a set of observable characteristics, $\mathbf{X_i}$, that might determine health outcomes, we run a number of placebo regressions of the following form, where x_i's denotes each element in the observable characteristics set $\mathbf{X_i}$:

$$x_i = \eta + \beta Downwind_i + \mu HighwayDistance_i + PUMA_i + Station_i + \varepsilon_i \tag{2}$$

The results of these regressions for a number of observable characteristics are reported in table S3, which present the coefficients and standard errors of our air quality instrument in regression models for each of the demographic characteristics included. We find few instances of significant correlations between our instrument and the various demographic characteristics that might impact COVID outcomes, most of them occurring in the Citywide and Outer Boroughs samples of tracts further away from highways (0.5 km to 1 km). For the sample of census tracts that lie between 0.05 and 0.5 km from the nearest highway, there is only one marginally significant correlation (at the 10% level) in the Citywide sample, and two in the Outer Boroughs sample. We see that there is an additional marginally significant correlation between per-capita income and our instrument in the 0-0.5 km sample, which is why our preferred model specifications use the Citywide sample of tracts that lie between 0.05 and 0.5 km from the nearest highway. These results give us confidence that our instrument allows us to identify the causal effect of ambient air quality on COVID outcomes in our analysis.

S3 Table: Placebo Checks for Instrument Correlation with Demographics

Highway Distance:	0 to 0.5 km		0.05 to 0.5 km		0.5 to 1 km	
Control Variables	Citywide	Outer Boroughs	Citywide	Outer Boroughs	Citywide	Outer Boroughs
Tract Population	601.80	1120.84	773.63	1208.55*	229.92	116.15
	(676.05)	(684.69)	(703.83)	(713.19)	(874.04)	(891.81)
% Change in 'Home' Devices	0.02	-0.02	0.02	-0.02	0.13	0.07
	(0.05)	(0.04)	(0.05)	(0.04)	(0.08)	(0.07)
% White	-0.03	-0.01	-0.05	-0.02	-0.09	-0.08
	(0.05)	(0.06)	(0.06)	(0.06)	(0.07)	(0.08)
% Black	-0.07*	-0.07	-0.07*	-0.08*	-0.09	-0.10
	(0.04)	(0.05)	(0.04)	(0.05)	(0.06)	(0.07)
Income Per Capita	8237.68*	7686.58*	5234.90	6035.62	-1592.73	1199.75
	(4726.80)	(4209.50)	(4802.76)	(4350.65)	(6889.25)	(6117.25)
% Aged 75+	-0.01	-0.01	-0.01	-0.01	0.00	0.00
	(0.01)	(0.01)	(0.01)	(0.01)	(0.01)	(0.01)
Median Tenure at Residence	-1.07	-0.95	-0.78	-0.94	-2.58*	-2.62*
	(1.18)	(1.23)	(1.23)	(1.29)	(1.32)	(1.43)
% of Residents with tenure >= 8 years	0.00	0.01	0.01	0.01	-0.02*	-0.02
	(0.01)	(0.01)	(0.01)	(0.01)	(0.01)	(0.01)
% Uninsured	-0.02	-0.03	-0.02	-0.02	0.05**	0.05**
	(0.02)	(0.02)	(0.02)	(0.02)	(0.02)	(0.02)
% Education: High School	-0.02	-0.02	-0.01	-0.01	0.01	0.01
	(0.02)	(0.02)	(0.02)	(0.02)	(0.03)	(0.03)
% Education: Bachelor's Degree	0.02	0.03	0.00	0.02	0.01	-0.01
	(0.02)	(0.02)	(0.02)	(0.02)	(0.03)	(0.03)
Gini Coefficient	0.00	0.00	0.00	0.01	0.02	0.02
	(0.02)	(0.02)	(0.02)	(0.02)	(0.02)	(0.03)

Notes: This table displays the results of our placebo checks described in equation 2. Coefficients of our air-quality instrument in regression models for each of the demographic characteristics calculated from the 2018 5-year ACS data and Safegraph data are presented with ***, **, and * indicating significance at the 1, 5, and 10 percent levels, respectively. Placebo checks were conducted for the entire city (Citywide) and for the Outer Boroughs sample, which includes tracts in Manhattan above 110th street, for tracts with the following distances to the nearest highway (Highway Distance): 0 to 0.5 km, 0.05 to 0.5 km, and 0.5 km to 1 km.

We then test the assumption that populations in our main sample (tracts between 0.05 and 0.5km of a highway) that spend relatively more time downwind of highways are similar to those that spend less time downwind by conducting balance checks comparing variables constructed from ACS data representing tract level socioeconomic and demo-

graphic characteristics. A tract is considered ‘Downwind’ if the amount of time the tract spends downwind of a highway is greater than the average amount of time tracts the same distance from a highway spend downwind, and is considered ‘Upwind’ otherwise.

Table S4 presents the results of the balance check conducted for tracts in our main sample with populations of at least 500 residents. For example, on average white residents make up 30.7% of the population for downwind tracts and 33.1% of the population for upwind tracts. The normalized difference between the two groups is 0.082. We show that no normalized differences between Upwind and Downwind tracts are greater than 0.25, supporting our assumption that conditional on living in a tract near a highway, there are not significant socioeconomic or demographic differences between tracts based on the amount of time they spend downwind of highways. While there are a number of reasons for residential sorting to occur in NYC, we believe it is unlikely that individuals are sorting into tracts based on the amount of time the tract spends downwind of a highway. For this reason, we assume that our instruments are exogenous and that the exclusion restriction is met.

S4 Table: Balance Checks

Balance Check for Census Tracts 0.05-0.5km of a Highway with Population > 500

5-Year 2018 ACS Variable	Downwind	Upwind	Normalized Difference
% White	0.307	0.331	-0.082
	[0.014]	[0.014]	
% Black/African American	0.161	0.186	-0.107
	[0.011]	[0.012]	
% Asian/Pacific Islander	0.163	0.163	-0.002
	[0.010]	[0.009]	
% Hispanic/Latinx	0.338	0.287	0.216
	[0.012]	[0.011]	
% Non-Latinx Other Race	0.032	0.033	-0.043
	[0.002]	[0.002]	
Tract Population	4,247.144	4,244.641	0.001
	[116.009]	[112.421]	
Per Capita Income	35,327.331	34,927.316	0.015
	[1353.746]	[1210.472]	
% Change in 'Home' Devices	-0.081	-0.083	0.016
	[0.008]	[0.007]	
Gini Coefficient	0.461	0.464	-0.06
	[0.003]	[0.003]	
% Aged Under 17	0.149	0.153	-0.093
	[0.002]	[0.002]	
% Aged 18-44	0.645	0.647	-0.046
	[0.003]	[0.002]	
% Aged 45-54	0.075	0.071	0.143
	[0.001]	[0.001]	
% Aged 55-64	0.064	0.062	0.087
	[0.001]	[0.001]	
% Aged 65-74	0.058	0.058	0.021
	[0.001]	[0.001]	
% Aged 75+	0.088	0.09	-0.06
	[0.001]	[0.001]	
% Uninsured	0.087	0.084	0.039
	[0.003]	[0.002]	
% Education: Bachelor's degree	0.214	0.212	0.025
	[0.005]	[0.005]	
% Education: Advanced degree	0.151	0.143	0.07
	[0.007]	[0.005]	
% Employed in Service Industry	0.237	0.233	0.043
	[0.006]	[0.005]	
% Employed in Production Industry	0.101	0.099	0.021
	[0.003]	[0.003]	
% Spending 50%+ of Income on Rent	0.299	0.309	-0.081
	[0.006]	[0.006]	

Notes: This table is a balance check of control variables from the 5-year 2018 ACS at the census tract level. Each census tract 0.05-0.5km from a highway was given a 'downwind' or 'upwind' designation. In this balance check, a census tract is considered to be 'downwind' if the amount of time a tract is downwind of a highway is greater than the mean time spent downwind for all tracts in this specification from 2008-2018, and is considered 'upwind' otherwise.

2.3 Second-Stage Regression

2.3.1 Log-linear model

Our second stage regressions estimate the causal impact of a change in ambient pollutant concentration on two different measures of the intensity of COVID-19 disease: deaths and hospitalizations. Given the discovery that rate-based measures of these outcomes, used to control for different populations across tracts, are subject to systematic measurement error which would lead to biased estimates in our regressions, we leave our dependent variables as counts. Thus our results can be interpreted as the change in deaths in an average tract, as our instruments are exogenous to tract population. Our key specification is:

$$\log Y_i = \alpha_0 + \alpha_1 \widehat{AQ}_i + \alpha_2 HighwayDistance_i + PUMA_i + Station_i + \nu_i \tag{3}$$

Y_i are counts of deaths and hospitalizations. We use log-transformed counts and add one to the count variables to adjust for tracts with zero deaths or hospitalizations. $\widehat{AQ}_i$ is the instrumented measure of ambient concentration for $PM_{2.5}$, NO_2, and NO predicted from equation (1). We include weather station and PUMA dummy variables in the second stage as well. The key parameter of interest is α_1. Our identification strategy compares tracts within the same PUMA that lie within the same distance of the highway that spent different amounts of time downwind from 2009-2018. We exclude census tracts with centroids located within 0.05km of the closest highway and with populations smaller than 500 as discussed above. There are 842 census tracts that fit all the above criteria in New York City.

We define two geographies for use in our analyses: *Citywide*, including all tracts across New York City, and *Outer Boroughs*, including all tracts in Brooklyn, the Bronx, Queens, and Staten Island, in addition to all tracts above 110th Street in Manhattan.

2.3.2 Poisson model

We also estimate a Poisson model using an instrumental variable control function estimator to avoid the incidental parameters problem from the large number of PUMA dummy variables (30-31). This method uses the residuals $\hat{\epsilon}_i$ estimated from equation (1) in the second stage:

$$Y_i = \mu_i \exp(\gamma_1 AQ_i + \gamma_2 \hat{\epsilon}_i + \gamma_3 HighwayDistance_i + Station_i) \exp(\nu_i) \quad (4)$$

In the control function approach, the residuals, $\hat{\epsilon}_i$, control for the endogenous portion of air quality. The PUMA dummies, μ_i are multiplicative. We estimate the coefficients using maximum likelihood.

3 Results and Discussions

3.1 Main Specifications

Our main specifications, presented in panel A of tables S5 and S6, are estimated with equation (3) using tracts with centroids that lie within 0.05-0.5 km of a highway and population greater than 500. Table S5 reports the coefficient estimates for our focal pollutants where the outcome variables are log transformed death counts. Table S6 reports the coefficient estimates for the log transformed number of hospitalizations as the outcome.

S5 Table: Effects of Air Pollution on Deaths

A. Deaths - log-linear (Main Specifications)

	(1)	(2)	(3)	(4)	(5)	(6)
$PM_{2.5}$	0.03	0.35				
	(0.69)	(0.93)				
NO_2			0.01	0.16		
			(0.31)	(0.43)		
NO					0.01	0.08
					(0.16)	(0.22)
Geography	Citywide	Outer Boroughs	Citywide	Outer Boroughs	Citywide	Outer Boroughs
N	842	751	842	751	842	751
Adjusted R^2	0.23	0.22	0.23	0.22	0.23	0.22

B. Deaths - OLS

	(1)	(2)	(3)	(4)	(5)	(6)
$PM_{2.5}$	-0.24^{*}	0.00				
	(0.14)	(0.17)				
NO_2			-0.11^{*}	0.01		
			(0.06)	(0.08)		
NO					-0.06^{*}	-0.01
					(0.03)	(0.04)
Geography	Citywide	Outer Boroughs	Citywide	Outer Boroughs	Citywide	Outer Boroughs
N	842	751	842	751	842	751
Adjusted R^2	0.23	0.22	0.23	0.22	0.23	0.22

C. Deaths - Poisson

	(1)	(2)	(3)	(4)	(5)	(6)
$PM_{2.5}$	0.13	0.32				
	(−1.32, 1.73)	(−1.64, 2.19)				
NO_2			0.06	0.15		
			(−0.66, 0.75)	(−0.77, 1.05)		
NO					0.03	0.08
					(−0.35, 0.38)	(−0.51, 0.59)
Geography	Citywide	Outer Boroughs	Citywide	Outer Boroughs	Citywide	Outer Boroughs
N	842	751	842	751	842	751

Notes: These tables report the estimated effects of various chronic ambient pollutant concentrations on COVID-19 deaths for 3 specifications: A. log-linear (main), B. OLS, and C. Poisson. In panels A. and B. of this table, robust standard errors are reported in parentheses and ***, **, and * indicate significance at the 1, 5, and 10 percent levels, respectively. Panel C. reports the estimated 95% confidence intervals in parentheses. These estimates are for all tracts with centroids that lie within 0.05-0.5km of a highway and population greater than 500 people.

S6 Table: Effects of Air Pollution on Hospitalizations

A. Hospitalization - log-linear (Main Specifications)

	(1)	(2)	(3)	(4)	(5)	(6)
$PM_{2.5}$	0.20	0.79				
	(0.67)	(0.89)				
NO_2			0.09	0.37		
			(0.31)	(0.41)		
NO					0.04	0.19
					(0.15)	(0.21)
Geography	Citywide	Outer Boroughs	Citywide	Outer Boroughs	Citywide	Outer Boroughs
N	842	751	842	751	842	751
Adjusted R^2	0.30	0.28	0.30	0.28	0.30	0.27

B. Hospitalizations - OLS

	(1)	(2)	(3)	(4)	(5)	(6)
$PM_{2.5}$	-0.31^{***}	-0.29^{*}				
	(0.12)	(0.16)				
NO_2			-0.12^{**}	-0.12		
			(0.05)	(0.08)		
NO					-0.07^{***}	-0.08^{**}
					(0.03)	(0.04)
Geography	Citywide	Outer Boroughs	Citywide	Outer Boroughs	Citywide	Outer Boroughs
N	842	751	842	751	842	751
Adjusted R^2	0.32	0.33	0.32	0.33	0.32	0.34

C. Hospitalizations - Poisson

	(1)	(2)	(3)	(4)	(5)	(6)
$PM_{2.5}$	0.52	1.00				
	(−0.82, 2.16)	(−0.72, 3.34)				
NO_2			0.24	0.46		
			(−0.42, 0.92)	(−0.33, 1.56)		
NO					0.12	0.24
					(−0.18, 0.47)	(−0.17, 0.78)
Geography	Citywide	Outer Boroughs	Citywide	Outer Boroughs	Citywide	Outer Boroughs
N	842	751	842	751	842	751

Notes: These tables report the estimated effects of various chronic ambient pollutant concentrations on COVID-19 hospitalizations for 3 specifications: A. log-linear (main), B. OLS, and C. Poisson. In panels A. and B. of this table, robust standard errors are reported in parentheses and ***, **, and * indicate significance at the 1, 5, and 10 percent levels, respectively. Panel C. reports the estimated 95% confidence intervals in parentheses. These estimates are for all tracts with centroids that lie within 0.05-0.5km of a highway and population greater than 500 people.

In tables S5 and S6, panel B estimates are from OLS models that do not account for the endogeneity of air pollution to contrast with the instrumental variable estimates in panel A. Panel C in tables S5 and S6 display the results estimated with Poisson models as specified in equation (4). 95% confidence intervals for coefficients in the Poisson model are bootstrapped with 1000 replications.

3.2 Challenges to Causal Identification

Studies consistently show that low-income and majority non-white neighborhoods are often subjected to higher ambient concentrations of air pollutants (12-16). These demographic characteristics also explain health outcomes (e.g., asthma rates). Using raw measures of ambient pollutant concentration will bias all coefficient estimates in regressions run on models seeking to explain health outcomes, meaning that these demographic characteristics can confound the results from recent observational studies.

Our instrumental variables approach allows us to identify the exogenous portion of air pollutant concentration, namely that which is not correlated with observable or unobservable demographic characteristics. That said, we acknowledge that census tracts near the highway will differ in meaningful ways from tracts further from the highway. To underscore the concerns about using endogenous measures of air quality, we apply our instrumental variables approach to all tracts that lie within 1km (as opposed to 0.5 km) of the nearest highway.

As shown in table S7 and S8, only when we run our instrumental variables regressions using the set of tracts within 1 km of the nearest highway are we able to replicate the large correlations between air pollution and COVID-19 outcomes that have been presented in observational studies. Recall from table S3 that our exogenous measures of ambient pollutant concentration are correlated with several demographic characteristics in the set of tracts that lie between 0.5 km and 1 km of the nearest highway, invalidating the instrument in this set of pixels. In other words, because the instruments in the 1 km sample are inevitably correlated with demographic characteristics, the instrumental variable approach is no longer valid, and the estimation suffers from an endogeneity bias, similar to observational studies.

TRAP concentrations decay quickly with distance from highways (32). For this reason,

and due to variation in demographic characteristics across census tracts as you move further from the nearest highway that invalidates our instrument, our preferred sample is the 0.05-0.5 km Citywide sample.

S7 Table: Effects of Air Pollution on Deaths: Tracts 0.5 km - 1 km from a Highway

A. $PM_{2.5}$

	(1)	(2)	(3)	(4)
$PM_{2.5}$	0.80	0.03	1.04*	0.35
	(0.51)	(0.69)	(0.61)	(0.93)
Geography	Citywide	Citywide	Outer Boroughs	Outer Boroughs
Max Dist	1km	0.5km	1km	0.5km
N	1,407	842	1,254	751
Adjusted R^2	0.15	0.23	0.16	0.22

B. NO_2

	(1)	(2)	(3)	(4)
NO_2	0.36	0.01	0.49*	0.16
	(0.23)	(0.31)	(0.29)	(0.43)
Geography	Citywide	Citywide	Outer Boroughs	Outer Boroughs
Max Dist	1km	0.5km	1km	0.5km
N	1,407	842	1,254	751
Adjusted R^2	0.14	0.23	0.16	0.22

C. NO

	(1)	(2)	(3)	(4)
NO	0.19	0.01	0.26*	0.08
	(0.12)	(0.16)	(0.15)	(0.22)
Geography	Citywide	Citywide	Outer Boroughs	Outer Boroughs
Max Dist	1km	0.5km	1km	0.5km
N	1,407	842	1,254	751
Adjusted R^2	0.14	0.23	0.15	0.22

Notes: These tables report the estimated effects of various chronic ambient pollutant concentrations on COVID-19 deaths for tracts with centroids that lie at a maximum of 0.5 or 1km from the nearest highway segment using our main log-linear specification. Robust standard errors are reported in parentheses and ***, **, and * indicate significance at the 1, 5, and 10 percent levels, respectively, and estimated 95% confidence intervals are reported below in parentheses. These estimates are for all qualifying tracts Citywide and in our Outer Borough sample that include at least 500 people, according to the 2018 5-year ACS.

S8 Table: Effects of Air Pollution on Hospitalizations:
Tracts 0.5 km - 1 km from a Highway

A. $PM_{2.5}$

	(1)	(2)	(3)	(4)
$PM_{2.5}$	1.15**	0.20	1.48**	0.79
	(0.52)	(0.67)	(0.63)	(0.89)
Geography	Citywide	Citywide	Outer Boroughs	Outer Boroughs
Max Dist	1km	0.5km	1km	0.5km
N	1,407	842	1,254	751
Adjusted R^2	0.16	0.30	0.20	0.28

B. NO_2

	(1)	(2)	(3)	(4)
NO_2	0.51**	0.09	0.68**	0.37
	(0.24)	(0.31)	(0.30)	(0.41)
Geography	Citywide	Citywide	Outer Boroughs	Outer Boroughs
Max Dist	1km	0.5km	1km	0.5km
N	1,407	842	1,254	751
Adjusted R^2	0.16	0.30	0.20	0.28

C. NO

	(1)	(2)	(3)	(4)
NO	0.26**	0.04	0.36**	0.19
	(0.12)	(0.15)	(0.16)	(0.21)
Geography	Citywide	Citywide	Outer Boroughs	Outer Boroughs
Max Dist	1km	0.5km	1km	0.5km
N	1,407	842	1,254	751
Adjusted R^2	0.16	0.30	0.18	0.27

Notes: These tables report the estimated effects of various chronic ambient pollutant concentrations on COVID-19 hospitalizations for tracts with centroids that lie at a maximum of 0.5 or 1km from the nearest highway segment using our main log-linear specification. Robust standard errors are reported in parentheses and ***, **, and * indicate significance at the 1, 5, and 10 percent levels, respectively, and estimated 95% confidence intervals are reported below in parentheses. These estimates are for all qualifying tracts City-wide and in our Outer Borough sample that include at least 500 people, according to the 2018 5-year ACS.

3.3 Age and Race-Ethnicity Regressions

We also explore whether the effects of long-term air pollution concentration on the intensity of COVID-19 disease differ based on demographics. To do so, we interact age and race-ethnicity group indicators with our instrumented measure of pollutant concentrations and estimate modified versions of equation (3). The outcome variables are log transformed counts of deaths and hospitalizations for each age and race-ethnicity group in each census tract. The race-ethnicity groups include White (W), Hispanic/Latino (H), Black/African American (B), and Asian/Pacific Islander (A). The age groups are under 18, 18-65, and 65+. Table S9 reports the results of the regressions with our age-instrument interactions and Table S10 reports the results of the regressions with our race-ethnicity-instrument interactions. The excluded categories are under 18 and White. We do not observe any meaningful heterogeneity by age group. While some of the interaction coefficients by race are significant or marginally significant, this could be due to chance as a result of testing so many hypotheses. In fact, when we correct the p-values for multiple hypothesis testing using the Benjamini and Hochberg (1995) false discovery rate method, none of the coefficients are significant at the 95% level, though the coefficient on the Hispanic/Latino interaction term has a p value of .07 for all three pollutants for hospitalizations, and .12 for deaths.

S9 Table: Effects of Air Pollution on Deaths and Hospitalizations: Age-Instrumented AQ Interaction Regressions

	Deaths - log-linear		
	(1)	(2)	(3)
$PM_{2.5}$	0.15		
	(0.28)		
NO_2		0.07	
		(0.13)	
NO			0.03
			(0.06)
age 18-64	1.07	1.07	1.06
	(2.27)	(2.27)	(1.39)
age 65+	1.45	1.45	1.58
	(2.48)	(2.49)	(1.52)
$PM_{2.5}$:age 18-64	−0.001		
	(0.24)		
$PM_{2.5}$:age 65+	0.04		
	(0.26)		
NO_2:age 18-64		−0.0003	
		(0.10)	
NO_2:age 65+		0.02	
		(0.11)	
NO:age 18-64			−0.0002
			(0.06)
NO:age 65+			0.01
			(0.07)
N	2,526	2,526	2,526
Adjusted R^2	0.65	0.65	0.65

	Hospitalizations - log-linear		
	(1)	(2)	(3)
$PM_{2.5}$	0.33		
	(0.36)		
NO_2		0.15	
		(0.16)	
NO			0.08
			(0.08)
age 18-64	1.41	1.41	1.70
	(2.99)	(3.01)	(1.83)
age 65+	2.21	2.21	2.15
	(2.73)	(2.74)	(1.67)
]$PM_{2.5}$:age 18-64	0.08		
	(0.32)		
$PM_{2.5}$:age 65+	−0.02		
	(0.29)		
NO_2:age 18-64		0.03	
		(0.14)	
NO_2:age 65+		−0.01	
		(0.13)	
NO:age 18-64			0.02
			(0.08)
NO:age 65+			−0.004
			(0.08)
N	2,526	2,526	2,526
Adjusted R^2	0.73	0.73	0.73

S10 Table: Effects of Air Pollution on Deaths and Hospitalizations: Race-Ethnicity and Age Instrumented-AQ Interaction Regressions

	Deaths - log-linear				Hospitalizations - log-linear		
	(1)	(2)	(3)		(1)	(2)	(3)
$PM_{2.5}$	−0.22			$PM_{2.5}$	−0.32		
	(0.47)				(0.61)		
NO_2		−0.09		NO_2		−0.13	
		(0.21)				(0.28)	
NO			−0.07	NO			−0.10
			(0.11)				(0.14)
Hispanic/Latino	-7.71^{*}	-7.73^{*}	-4.60^{*}	Hispanic/Latino	-11.93^{**}	-11.96^{**}	-7.13^{**}
	(3.95)	(4.00)	(2.39)		(5.11)	(5.16)	(3.06)
Black	−0.08	−0.08	−0.09	Black	−3.10	−3.11	−1.95
	(3.72)	(3.72)	(2.24)		(4.84)	(4.85)	(2.92)
Asian/Pacific Islander	-6.92^{*}	-6.94^{*}	-4.39^{**}	Asian/Pacific Islander	−7.08	−7.09	-4.56^{*}
	(3.60)	(3.57)	(2.17)		(4.56)	(4.57)	(2.76)
$PM_{2.5}$:Hispanic/Latino	0.84^{**}			$PM_{2.5}$:Hispanic/Latino	1.30^{**}		
	(0.42)				(0.54)		
$PM_{2.5}$:Black	−0.005			$PM_{2.5}$:Black	0.31		
	(0.39)				(0.51)		
$PM_{2.5}$:Asian/Pacific Islander	0.69^{*}			$PM_{2.5}$:Asian/Pacific Islander	0.68		
	(0.38)				(0.48)		
NO_2:Hispanic/Latino		0.37^{**}		NO_2:Hispanic/Latino		0.57^{**}	
		(0.18)				(0.24)	
NO_2:Black		−0.002		NO_2:Black		0.14	
		(0.17)				(0.22)	
NO_2:Asian/Pacific Islander		0.30^{*}		NO_2:Asian/Pacific Islander		0.30	
		(0.16)				(0.21)	
NO:Hispanic/Latino			0.23^{**}	NO:Hispanic/Latino			0.35^{**}
			(0.11)				(0.14)
NO:Black			−0.001	NO:Black			0.08
			(0.10)				(0.13)
NO:Asian/Pacific Islander			0.18^{*}	NO:Asian/Pacific Islander			0.18
			(0.10)				(0.13)
N	3,368	3,368	3,368	N	3,368	3,368	3,368
Adjusted R^2	0.04	0.05	0.07	Adjusted R^2	0.07	0.07	0.09

3.4 Count-Based vs Rate-Based Measures

Most previous studies on the relationship between air quality and COVID-19 outcomes have normalized the outcomes by population. However, given the discussion surrounding Figure 4 showing that actual tract population differs from that available in existing administrative data, we believe that rate-based measures reliant on administrative data are inappropriate measures of COVID-19 outcomes, given the number of people who left the city in some tracts. To further explore the shortcomings of rate-based measures in this context, we show results from OLS models using rate-based dependent variables in Table S11. Table S11 shows the results of regressions on mortality rates (Deaths/100,000) using an OLS approach. We also use tract population weights, and populations adjusted according to the Safegraph data in these specifications.

Table 11 panel A shows results from the OLS model with no control variables, panel B displays results with control variables. Panels C and D show results from the OLS models with control variables and population weights. Panel C weights for tract population, and panel D displays results from OLS models using the Safegraph adjusted measure of rates. We adjust census tract populations by the fraction of Safegraph devices that left each home census tract between weeks 10 and 20 of 2020. This adjustment is likely unsatisfactory because older individuals are less likely to be represented in the Safegraph data and more likely to be susceptible to COVID-19 and poor air quality, but it does illustrate the challenge posed by rate-based outcome measures.

S11 Table: Effects of Air Pollution on Deaths:
Citywide Results Using Rate Based Measures

A. OLS

	(1)	(2)	(3)
PM2.5	12.97		
	(22.37)		
NO2		10.56	
		(9.43)	
NO			6.68
			(5.02)
Controls	No	No	No
N	842	842	842
Adjusted R^2	0.14	0.14	0.14

B. OLS w/ Controls

	(1)	(2)	(3)
PM2.5	33.05		
	(22.91)		
NO2		14.71	
		(9.78)	
NO			8.03
			(4.96)
Controls	Yes	Yes	Yes
N	840	840	840
Adjusted R^2	0.28	0.28	0.28

S11 Table: Effects of Air Pollution on Deaths:
Citywide Results Using Rate Based Measures (continued)

C. Population Weighted OLS w/ Controls

	(1)	(2)	(3)
PM2.5	1.93		
	(16.41)		
NO2		2.07	
		(6.94)	
NO			0.56
			(3.61)
Controls	Yes	Yes	Yes
N	840	840	840
Adjusted R^2	0.33	0.33	0.33

D. Adj. Population Weighted OLS w/ Controls

	(1)	(2)	(3)
PM2.5	0.42		
	(21.70)		
NO2		1.19	
		(9.39)	
NO			−0.09
			(4.85)
Controls	Yes	Yes	Yes
N	839	839	839
Adjusted R^2	0.24	0.24	0.24

Notes: The above tables showcase the citywide sample results from OLS models using long-term averages of TRAP pollutants as the independent variables and the number of deaths per 100,000 residents as the dependant variable. Panel A shows results from this simple OLS model with no control variables. Panel B displays results from the OLS models with control variables. Panel C shows the results from OLS models with control variables and weighted for population, and Panel D results are from OLS models using the Safegraph adjusted measure of rates, with control variables and Safegraph-adjusted population weights. Robust standard errors are reported in parentheses and ***, **, and * indicate significance at the 1, 5, and 10 percent levels, respectively.

3.5 Demographic Controls

In addition to the robustness checks previously mentioned, we run versions of our main specification equation (3) with demographic control variables from ACS and Safegraph. This runs the risk of biasing estimation by conditioning on post-treatment variables, as air quality has been shown to affect educational achievement and income, for instance (33). All of our key estimates become negative, though small and statistically insignificant as shown in Table S12.

S12 Table: Effects of Air Pollution on Deaths: IV with Demographics Controls

	Deaths - log-linear - Demographic Controls					
	(1)	(2)	(3)	(4)	(5)	(6)
PM2.5	−0.54	−0.49				
	(0.57)	(0.81)				
NO2			−0.24	−0.21		
			(0.25)	(0.35)		
NO					−0.12	−0.11
					(0.13)	(0.18)
Log Tract Population	0.74***	0.76***	0.74***	0.76***	0.74***	0.75***
	(0.05)	(0.05)	(0.05)	(0.05)	(0.05)	(0.05)
Log Income Per Capita	−0.06	−0.11	−0.07	−0.11	−0.08	−0.12
	(0.15)	(0.17)	(0.15)	(0.17)	(0.15)	(0.17)
% Owner Occupied	−0.26*	−0.34*	−0.25	−0.33*	−0.24	−0.32*
	(0.16)	(0.19)	(0.15)	(0.18)	(0.15)	(0.17)
% Received Public Assistance	−0.87	−0.95	−0.80	−0.90	−0.88	−0.96
	(0.64)	(0.65)	(0.63)	(0.65)	(0.64)	(0.66)
% Rent-Income > 50%	−0.13	−0.16	−0.14	−0.17	−0.15	−0.18
	(0.21)	(0.22)	(0.21)	(0.22)	(0.21)	(0.22)
% Education: High School	0.68*	0.58	0.71*	0.61	0.70*	0.60
	(0.37)	(0.39)	(0.36)	(0.38)	(0.36)	(0.39)
% Education: less than high school	0.64*	0.46	0.69*	0.51	0.64	0.45
	(0.39)	(0.46)	(0.39)	(0.44)	(0.39)	(0.46)
% Black/African American	0.45***	0.42***	0.44***	0.42***	0.45***	0.42***
	(0.15)	(0.16)	(0.15)	(0.16)	(0.15)	(0.16)
% Hispanic/Latino	0.39**	0.41**	0.37**	0.39**	0.39**	0.41**
	(0.16)	(0.18)	(0.16)	(0.17)	(0.16)	(0.18)
% Age 18-44	−0.04	0.21	−0.03	0.20	−0.06	0.18
	(0.53)	(0.56)	(0.53)	(0.55)	(0.53)	(0.56)
% Age 45-54	−3.22***	−2.84***	−3.19***	−2.84***	−3.26***	−2.87***
	(0.88)	(0.90)	(0.90)	(0.90)	(0.87)	(0.89)
% Age 55-64	−1.85*	−1.79*	−1.75*	−1.69*	−1.86*	−1.81*
	(0.99)	(1.01)	(1.02)	(1.03)	(0.98)	(1.01)
% Age 65-74	−4.09***	−4.79***	−4.09***	−4.80***	−4.12***	−4.80***
	(1.08)	(1.13)	(1.08)	(1.12)	(1.08)	(1.12)
% Age 75+	−2.85***	−3.11***	−2.93***	−3.18***	−2.90***	−3.16***
	(0.89)	(0.95)	(0.90)	(0.96)	(0.90)	(0.97)
Safegraph Device Change	0.08	0.08	0.10	0.10	0.09	0.08
	(0.15)	(0.17)	(0.14)	(0.16)	(0.15)	(0.17)
Geography	Citywide	Outer Boroughs	Citywide	Outer Boroughs	Citywide	Outer Boroughs
N	840	749	840	749	840	749
Adjusted R^2	0.55	0.55	0.55	0.55	0.56	0.55

Notes: This table reports the results from our main model specification while controlling for demographic characteristics of census tract populations calculated using 2018 5-year ACS data. Robust standard errors are reported in parentheses and ***, **, and * indicate significance at the 1, 5, and 10 percent levels, respectively. These estimates are for all qualifying tracts City-wide and in our Outer Borough sample that include at least 500 people, according to the 2018 5-year ACS.

3.6 Additional Robustness Checks

Table S13 reports additional robustness checks. Panel A reports the estimates from equation (3) using 100 residents rather than 500 residents as an alternative cutoff for tract population. Panel B reports the estimates from equation (3) using pollution interpolated from the closest monitor rather than inverse distance weighted averages. Panel C reports the estimates using the average distance to four nearest highway segments as "near dist" measure. Panel D displays the estimates using 30 degree as the cutoff for a more stringent measure of % of time downwind. Panel E reports the estimates trimming the top 5% and bottom 5% of tracts by population. All of our estimates are small and statistically insignificant as shown in Table S13.

S13 Table: Additional Robustness Checks

A. Deaths - log-linear - population cutoff 100

	(1)	(2)	(3)	(4)	(5)	(6)
$PM_{2.5}$	−0.04	0.23				
	(0.68)	(0.94)				
NO_2			−0.02	0.11		
			(0.31)	(0.43)		
NO					−0.01	0.06
					(0.16)	(0.22)
Geography	Citywide	Outer Boroughs	Citywide	Outer Boroughs	Citywide	Outer Boroughs
N	811	726	811	726	811	726
Adjusted R^2	0.23	0.22	0.23	0.22	0.23	0.22

B. Deaths - log-linear - pollution measured at closest monitor

	(1)	(2)	(3)	(4)	(5)	(6)
$PM_{2.5}$	0.02	0.33				
	(0.55)	(0.86)				
NO_2			0.01	0.15		
			(0.24)	(0.40)		
NO					0.01	0.10
					(0.14)	(0.27)
Geography	Citywide	Outer Boroughs	Citywide	Outer Boroughs	Citywide	Outer Boroughs
N	842	751	842	751	842	751
Adjusted R^2	0.28	0.26	0.28	0.26	0.28	0.25

C. Deaths - log-linear - alternative measure of distance to nearest highway

	(1)	(2)	(3)	(4)	(5)	(6)
$PM_{2.5}$	−0.16	0.03				
	(0.61)	(0.82)				
NO_2			−0.07	0.02		
			(0.28)	(0.38)		
NO					−0.04	0.01
					(0.14)	(0.20)
Geography	Citywide	Outer Boroughs	Citywide	Outer Boroughs	Citywide	Outer Boroughs
N	832	746	832	746	832	746
Adjusted R^2	0.24	0.23	0.24	0.23	0.24	0.23

S13 Table: Additional Robustness Checks (continued)

D. Deaths - log-linear - downwind defined with 30 degrees

	(1)	(2)	(3)	(4)	(5)	(6)
$PM_{2.5}$	0.14	0.46				
	(0.61)	(0.81)				
NO_2			0.06	0.21		
			(0.27)	(0.37)		
NO					0.03	0.11
					(0.14)	(0.19)
Geography	Citywide	Outer Boroughs	Citywide	Outer Boroughs	Citywide	Outer Boroughs
N	842	751	842	751	842	751
Adjusted R^2	0.22	0.21	0.22	0.21	0.22	0.21

E. Deaths - log-linear - top and bottom 5% windsorized by tract population

	(1)	(2)	(3)	(4)	(5)	(6)
$PM_{2.5}$	0.42	0.70				
	(0.85)	(1.05)				
NO_2			0.20	0.34		
			(0.41)	(0.51)		
NO					0.10	0.18
					(0.21)	(0.27)
Geography	Citywide	Outer Boroughs	Citywide	Outer Boroughs	Citywide	Outer Boroughs
N	781	699	781	699	781	699
Adjusted R^2	0.21	0.19	0.20	0.19	0.20	0.18

Notes: Additional robustness checks using equation (3) are reported above as follows: Panel A displays estimates using 100 rather than 500 as the cutoff for tract population, panel B reports the estimates using pollution interpolated from the closest monitor rather than inverse distance weighted averages, panel C reports the estimates using the average distance of 4 nearest highway segments as "near dist" measure, panel D displays the estimates using 30 degree as the cutoff for a more stringent measure of % of time downwind, and panel E reports the estimates that result from trimming the top and bottom 5% of tracts by population. Robust standard errors are reported in parentheses and ***, **, and * indicate significance at the 1, 5, and 10 percent levels, respectively.

4 Supplement References

1. Anderson ML. As the wind blows: The effects of long-term exposure to air pollution on mortality. Journal of the European Economic Association. 2020;18(4):1886-1927.

2. Benjamini Y, Hochberg Y. Controlling the false discovery rate: a practical and powerful approach to multiple testing. Journal of the Royal statistical society: series B (Methodological) 57, no. 1 (1995): 289-300.

3. Deryugina T, Heutel G, Miller NH, Molitor D, Reif J. The mortality and medical costs of air pollution: Evidence from changes in wind direction. American Economic Review. 2019;109(12):4178-4219.

4. Fowlie M, Rubin E, Walker R. Bringing satellite-based air quality estimates down to earth. AEA Papers and Proceedings. 2019;109:283-288.

5. Krupnick A. Satellites Can Supplement the Clean Air Act's Land-Based Air Monitoring Network. Resources. 2020;204.

6. Grainger C, Schreiber A, Chang W. Do Regulators Strategically Avoid Pollution Hotspots when Siting Monitors? Evidence from Remote Sensing of Air Pollution. Revise and Resubmit, American Economics Journal: Economic Policy. 2020.

7. Grainger C, Schreiber A. Discrimination in ambient air pollution monitoring? AEA Papers and Proceedings. 2019;109:277-282.

8. Schlenker W, Walker WR. Airports, air pollution, and contemporaneous health. Review of Economic Studies. 2016;83(2):768-809.

5 Software References

1. Angelo Canty and Brian Ripley (2021). boot: Bootstrap R (S-Plus) Functions. R package version 1.3-27.

2. Baptiste Auguie (2017). gridExtra: Miscellaneous Functions for "Grid" Graphics. R package version 2.3. https://CRAN.R-project.org/package=gridExtra

3. Berge L (2018). "Efficient estimation of maximum likelihood models with multiple fixed-effects: the R package FENmlm." CREA Discussion Papers.

4. Christian Kleiber and Achim Zeileis (2008). Applied Econometrics with R. New York: Springer-Verlag. ISBN 978-0-387-77316-2. https://CRAN.R-project.org/package=AER

5. Hadley Wickham and Evan Miller (2019). haven: Import and Export 'SPSS', 'Stata' and 'SAS' Files. R package version 2.2.0.

6. Hlavac, Marek (2018). stargazer: Well-Formatted Regression and Summary Statistics Tables. R package version 5.2.1. https://CRAN.R-project.org/package=stargazer

7. Hyunseung Kang, Yang Jiang, Qingyuan Zhao and Dylan Small (2021). ivmodel: Statistical Inference and Sensitivity Analysis for Instrumental Variables Model. R

8. Pebesma, E., 2018. Simple Features for R: Standardized Support for Spatial Vector Data. The R Journal 10 (1), 439-446, https://doi.org/10.32614/RJ-2018-009

9. Zeileis A (2006). "Object-Oriented Computation of Sandwich Estimators." Journal of Statistical Software, *16*(9), 1-16. doi: 10.18637/jss.v016.i09

10. Wickham et al., (2019). Welcome to the tidyverse. Journal of Open Source Software, 4(43), 1686, https://doi.org/10.21105/joss.01686
